# Hypoxia-induced exosomal LUCAT1 promotes osimertinib resistance in lung adenocarcinoma by stabilizing c-MET

**DOI:** 10.1038/s41419-025-08100-2

**Published:** 2025-10-27

**Authors:** Jianting Du, Bin Zheng, Jiekun Qian, Guanglei Huang, Wenjie Yuan, Renjie Huang, Xian Gong, Guobing Xu, Bixing Zhao, Xiaolong Liu, Yingchao Wang, Zhang Yang, Chun Chen

**Affiliations:** 1 Department of Thoracic Surgery, Fujian Medical University Union Hospital, Fuzhou, Fujian China; 2 Key Laboratory of Cardio-Thoracic Surgery, Fujian Medical University, Fuzhou, Fujian China; 3 Clinical Research Center for Thoracic Tumors of Fujian Province, Fuzhou, Fujian China; 4 The United Innovation of Mengchao Hepatobiliary Technology Key Laboratory of Fujian Province, Mengchao Hepatobiliary Hospital of Fujian Medical University, Fuzhou, Fujian China

**Keywords:** Non-small-cell lung cancer, Predictive markers

## Abstract

Osimertinib resistance poses a major challenge in treating advanced EGFR-mutant lung adenocarcinoma (LUAD). Exosomes, key mediators of intercellular communication, may contribute to drug resistance, but their specific role in osimertinib resistance remains unclear. This study aimed to elucidate the function and mechanisms of hypoxia-induced exosomes (HExo) in promoting osimertinib resistance in LUAD, with the goal of identifying potential diagnostic biomarkers and therapeutic targets. Cell survival under osimertinib treatment was analyzed using CCK8 and colony formation assays, while exosomal LUCAT1 was identified via RNA-seq and validated by qRT-PCR. Biological roles of LUCAT1 were assessed through in vitro and in vivo experiments, including immunoblotting, RNA immunoprecipitation (RIP)-qPCR, xenograft tumor models, and organoid models. Results demonstrated that hypoxia-induced exosomal LUCAT1 reduced the sensitivity of recipient LUAD cells to osimertinib. Mechanistically, LUCAT1 promoted osimertinib resistance by preventing c-MET degradation and activating downstream AKT/mTOR and ERK pathways. Targeting c-MET effectively restored osimertinib sensitivity in resistant cells. Moreover, higher levels of exosomal LUCAT1 were significantly associated with poor therapeutic responses to osimertinib in patients. In conclusion, hypoxia-induced exosomal LUCAT1 drives osimertinib resistance by stabilizing c-MET and activating its downstream pathways. Plasma exosomal LUCAT1 levels are closely linked to osimertinib resistance and may serve as an ideal liquid biopsy target for monitoring patient response.

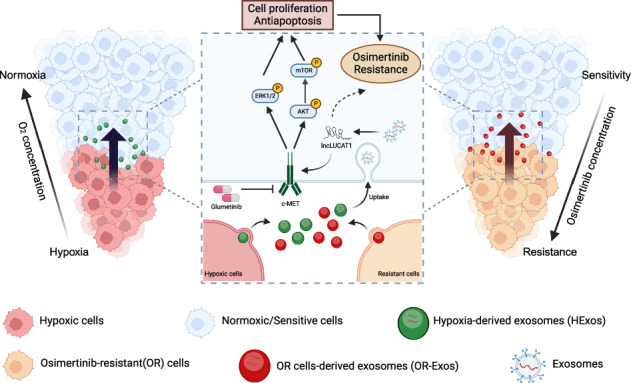

## Introduction

Lung cancer (LUCA) is a notorious malignancy worldwide [1], and lung adenocarcinoma (LUAD), as a subtype of non-small cell lung cancer (NSCLC), is the most prevalent histological subtype, accounting for approximately 40% of lung malignancies [2]. More importantly, the prognosis of patients with advanced-stage LUAD is poor, and the 5-year overall survival (OS) rate is less than 20% [3].

Since the identification of driver oncogenes in 2002, targeted therapy has emerged as a promising strategy for treating advanced LUAD. However, drug resistance often emerges, limiting the long-term efficacy of these treatments [4]. Epidermal growth factor receptor (EGFR) mutation, a frequent mutation in LUAD, has been particularly challenging in this regard [5]. Osimertinib, a third-generation EGFR-tyrosine kinase inhibitor (TKI), has shown remarkable efficacy by selectively and irreversibly targeting both sensitive EGFR mutations (19del and L858R) and the resistant T790M mutation. Despite its initial success, resistance to osimertinib invariably occurs in all treated patients [6]. The FLAURA study identified mesenchymal to epithelial transition factor (c-MET) amplification as the most common cause of resistance in patients receiving osimertinib as the first-line therapy, accounting for 15% of cases, followed by the EGFR C797S mutation [7]. Similarly, the AURA3 study highlighted c-MET amplification as the predominant resistance mechanism in the second-line osimertinib treatment [8, 9]. However, the precise mechanisms by which c-MET amplification or protein overexpression drives osimertinib resistance remain unclear, and no effective treatment strategies are currently available to extend OS for patients with acquired resistance. Therefore, identifying new biomarkers and treatment strategies is essential.

Exosomes are microscopic extracellular vesicles secreted by cells, consisting of a lipid bilayer and typically measuring 30–150 nm in diameter. Increasing evidence revealed that exosomes play critical roles in cell-to-cell communication through transmitting signals to recipient cells and alter their behaviors [10, 11]. Our previous research demonstrated that hypoxia-induced exosomes (HExos) can transport microRNAs (miRNAs), enhance the invasiveness and metastasis of recipient tumor cells [12]. Additional studies have shown that exosomes secreted by tumor cells contribute to cancer progression by reshaping the tumor microenvironment, degrading the extracellular matrix, increasing cell invasiveness and metastasis, mediating immune suppression, and facilitating resistance to radiotherapy and chemotherapy [13–15]. Given these roles, exosomes hold significant promise as biomarkers for cancers.

Long non-coding RNAs (lncRNAs) are defined as a class of RNA molecules longer than 200 nucleotides that lack protein-coding capacity [16]. Remarkably, more than 90% of the transcripts in the human genome fall into this category [17]. In recent years, accumulating evidence demonstrated that numerous lncRNAs are abnormally expressed in malignant tumors, and their biological functions span nearly all aspects of tumor cell activities, including cell proliferation, apoptosis, angiogenesis, and drug resistance [18, 19]. Notably, LUCAT1, a well-studied lncRNA, has been linked to chemotherapy resistance across several cancer types [20, 21]. However, it remains unclear whether LUCAT1, especially exosomal LUCAT1, participates in targeted drug resistance of tumor cells. In this study, we found that hypoxia-induced exosomal LUCAT1 promoted the osimertinib resistance of LUAD cells with EGFR mutation by interacting with and stabilizing c-MET for the first time. Furthermore, exosomal LUCAT1 was also identified by us as a promising diagnostic biomarker and therapeutic target for osimertinib resistance in LUAD.

## Materials and methods

### Study design

This study systematically investigated the role of exosomal LUCAT1 in mediating osimertinib resistance in EGFR-mutant LUAD through a stepwise experimental workflow (as shown in Supplementary Fig. S1A). Briefly, exosomes derived from hypoxic and normoxic cells were isolated and co-cultured with LUAD cells to assess their impact on osimertinib resistance. Subsequently, RNA sequencing identified differential expression of LUCAT1, followed by mechanistic validation through its overexpression/knockdown. Finally, LUAD organoid models were used to confirm that exosomes derived from drug-resistant cells could disseminate resistance phenotypes.

### Cell lines and reagents

Human NSCLC cell lines, including H1975 (EGFR L858R and T790M combined mutation) and HCC827 (EGFR exon 19 deletion) were obtained from the American Tissue culture collection (ATCC, VA, USA). The cells were maintained in RPMI-1640 medium containing 10% fetal bovine serum (FBS) and antibiotics (100 U/ml penicillin and 100 µg/ml streptomycin) in a humidified environment with 5% CO_2_ at 37 °C. Cells used for exosome extraction were all cultured in exosome-depleted serum-free media. All cells were authenticated by STR profiling and tested for mycoplasma contamination. For hypoxic treatment, the cells were cultured in a H35 HEPA hypoxystation (Whitley, UK) flushed with a mixture of 1% O_2_, 94% N_2_, and 5% CO_2_ for 48 h. The osimertinib-resistant (OR) cells were developed from H1975 and HCC827 cells by stepwise exposure to increasing concentrations of osimertinib from 20 nM to 10 μM, and the drug-resistant cell lines H1975/OR and HCC827/OR were established after 6 months.

Osimertinib and glumetinib were purchased from Selleck Chemicals (China). Cycloheximide, MG132, GW4869, RNase A, and Triton X-100 were purchased from MedchemExpress (China).

### Patient samples

A cohort of 30 LUAD tissues and plasma specimens was collected by biopsy or operation from advanced LUAD patients at Fujian Medical University Union Hospital (Fuzhou, China). The sample size was determined based on preliminary data and the sample size design of comparable studies. All plasma samples from LUAD patients were collected one day before initial treatment, including 10 patients with advanced LUAD who received osimertinib treatment. Detailed clinical data of these patients are listed in Supplementary Table S2. In addition, a sample of human blood plasma from 15 healthy donors (HDs) was also collected.

### Culture of EGFR-mutant LUAD organoids

Tumor specimens were finely disintegrated into small fragments (0.5–1 mm^3^) and incubated with a digestion solution containing 0.125 mg/ml collagenase IV (Sigma, USA) and 0.1 mg/ml DNase (Sigma, USA) at 37 °C for 0.5–1 h. After washing the pellet in cold advanced DMEM/F12 (GIBCO, USA), the pellet was mixed with Matrigel (CORNING, USA). A total of 3 × 10^3^–5 × 10^3^ cells were then seeded into pre-warmed 24-well plates. After solidification of Matrigel (~15–20 min), organoid culture medium was added to the cells. The organoids were subsequently cultured in an incubator maintained at 37 °C, 5% CO_2_, and >90% humidity. Organoid medium contained advanced DMEM/F12 supplemented with 1% penicillin/streptomycin (GIBCO, USA), 1% glutamax (GIBCO, USA), 10 mM HEPES (GIBCO, USA), 1:50 B27 supplement (GIBCO, USA), 1:100 N2 supplement (GIBCO, USA), 1.25 mM N-acetyl-L-cysteine (Sigma, USA), 10 mM nicotinamide (Sigma, USA), 50 ng/ml recombinant human epidermal growth factor (Peprotech, USA), 100 ng/ml recombinant human fibroblast growth factor 10 (Peprotech, USA), 25 ng/ml recombinant human hepatic growth factor (HGF) (Peprotech, USA), 10 μM forskolin (Selleck Chemicals, USA), and 5 μM A8301 (Selleck Chemicals, USA). The culture medium was changed twice a week. Organoids passaged at 80–90% confluency or diameter >100 μm (typically every 7–10 days), split at 1:3 ratio. All organoid cultures were tested for mycoplasma contamination every three months using a Lookout Mycoplasma PCR Detection Kit (Sigma, USA). Batch validation via morphology (H&E staining) and lineage markers (e.g., TTF-1/CK7 for LUAD organoids). For each randomly selected case of EGFR-mutant LUAD organoids, three biological replicates were set up. All experiments used organoids within ≤5 passages to prevent genetic drift or functional decline. Post-thaw organoids were limited to ≤3 passages.

### Cell transfection

We constructed the LUCAT1 pcDNA3.1 [OE (overexpression)-LUCAT1], inserting the respective genes into the pcDNA3.1 vector (Invitrogen). An empty pcDNA3.1 vector (OE-ctrl) was used as a control. For RNA interference (RNAi), the chemically synthesized small interfering RNA (siRNA) of c-MET was purchased from Sigma. All sh-LUCAT1 and sh-ctrl lentiviral vectors were obtained from GeneChem Co., Ltd, China. Supplementary Table S3 lists the sequences of relative siRNAs and short hairpin RNAs (shRNA). For detailed protocol, please refer to our previous articles [22].

### In vivo xenograft and treatment experiments

In all, 5–6 week old male NCG mice (GemPharmatech Co., China) were purchased and fed in standard pathogen-free conditions. A total of 3 × 10^6^ cells were injected subcutaneously into the flank of NCG mice. Xenograft volumes were evaluated by caliper every 3 days and calculated individually as the formula: Volume = 0.5 × length × width^2^. When tumor volume reached about 200 mm^3^, mice injected with the same cell type were randomly allocated to treatment groups for subsequent interventions in the same proportion. Then, 10 μg (100 μl) of HExos or 100 μl of PBS was injected into the tumor once daily for 14 consecutive days. On the 2nd day after HExos injection, intragastric administration of 5 mg/kg osimertinib begins and continues until the mice were euthanized. In addition, to investigate the effect of LUCAT1, we inoculated stable LUCAT1 overexpression or knockdown cells and control cells into the flanks of NCG mice. When tumor volume reached 200 mm^3^, mice bearing tumors were randomly assigned to four groups (each group with five mice) and osimertinib (a dose of 5 mg/kg) or Glumetinib (a dose of 5 mg/kg) treatment was administered by oral gavage every day. Experimental endpoints were defined as tumor volume exceeding 2000 mm³, body weight loss >15%, or severe morbidity (e.g., lethargy, ulceration). Animals meeting endpoint criteria were euthanized via cervical dislocation under isoflurane anesthesia.

### Exosome isolation, purification, and characterization

Exosomes were isolated from cell culture supernatant using ultracentrifugation (Beckman Coulter Optima XPN-100 ultracentrifuge, 100,000×*g* for 2 h at 4 °C) as previously described [12]. Then, the surface morphology and ultrastructure of exosomes from cell supernatants were analyzed by means of transmission electron microscopy (TEM). Nanoparticle tracking analysis (NTA) was performed to calculate exosome size distribution and concentration using Zetaview (Particle Metrix) equipped with fast video capture according to the manufacturer’s instructions. In addition, exosome-specific proteins (CD63, CD81, and TSG101) were identified using western blotting.

### RNA sequence

HExos and normoxia-induced exosomes (NExos) were collected for RNA sequencing. RNA was extracted from exosomes using Trizol reagent (Thermo). The extracted RNA was then quantified and checked for purity using a Nanodrop spectrophotometer and a Qubit fluorometer, ensuring accurate measurement of RNA concentration. The RNA was used for stranded RNA sequencing library preparation using Ribo-off rRNA Depletion Kit (Vazyme, China) to deplete ribosomal RNA (rRNA), and then components of KC-Digital Stranded mRNA Library Prep Kit (Wuhan Seqhealth Co., Ltd., China) following the manufacturer’s instructions. The kit eliminates duplication bias in PCR and sequencing steps by using a unique molecular identifier (UMI) of 12 random bases to label the pre-amplified cDNA molecules. The library products corresponding to 200–500 bp were enriched, quantified, and finally sequenced on Novaseq 6000 (Illumina) with PE150 mode.

### Protein stability

To determine the stability of c-MET, the translations of genes were stopped by cycloheximide (CHX, 50 μg/ml, Selleck, China) in LUCAT1-overexpressing and control H1975 and HCC827 cells. Cells were lysed by RIPA buffer supplemented with 1% protease inhibitor cocktails at the indicated time periods. And the expressions of c-MET were detected by western blot assay.

### Quantitative real-time polymerase chain reaction (qRT-PCR) analysis

Total RNA was extracted with the TransZol Up RNA kit (TransGen, China), while exosomal RNAs were obtained with the exoRNeasy Maxi Kit (Qiagen, CA), following the manufacturer’s instructions. The SYBR Green PCR Kit (TaKaRa, Japan) was used to amplify the cDNA templates for PCR. qRT-PCR was performed on an Applied Biosystems 7500 Sequence Detection System (Applied Biosystems, USA) with the following thermal cycling conditions: an initial step at 95 °C for 30 s, followed by 45 cycles of 95 °C for 5 s and 60 °C for 30 s, with a final extension at 72 °C for 5 minutes. The specific primers are detailed in Supplementary Table S4. Each experiment was conducted in triplicate, and the relative expression of RNA was calculated using 2^-ΔΔCt^ method.

### Cell counting Kit 8 (CCK-8) and colony-formation assays

Cell viability was measured by CCK-8 (GLPBIO, USA) according to the manufacturer’s instructions. We seeded 5000 cells with the corresponding treatment onto 96-well culture plates. At predetermined time intervals, CCK-8 reagent was added for incubation for 2 h at 37 °C. Then, the absorbance at 450 nm was determined by a Multi-Mode Microplate Reader (Molecular Devices, USA).

For colony-formation assays, cells were seeded in a six-well plate (1000 cells per well) and cultured with complete medium for 14 days. Following seeding, the medium containing drugs was replaced every three days. Cell colonies were fixed with 4% paraformaldehyde (Beyotime, China) for 30 minutes and stained with crystal violet (Beyotime, China). Photographs were captured, and the colonies were quantified using ImageJ software.

### RNA immunoprecipitation (RIP)-qPCR assay

RIP was implemented using a Magna RIP™RNA-Binding Protein Immunoprecipitation Kit (Millipore, USA) as directed by the manufacturer. Briefly, the lysates of 2 × 10^7^ H1975 or HCC827 cells were incubated with beads pre-coated with IgG or antibody against c-MET (Cell Signaling Technology, USA) overnight at 4 °C in the presence of 2U/ml RNasin (Promega, USA). The incubated beads were washed with 500 μL RIP wash buffer by transient vortex for six times. Afterwards, the beads were resuspended in proteinase K buffer including 10 mg/mL proteinase K and 10% SDS, and incubated at 55 °C for 30 min. The immunoprecipitated RNAs were then extracted with the mixture (phenol: chloroform: isoamylol = 25:24:1) by centrifuging at 14,000 rpm for 10 min at room temperature, and purified with ethanol including 0.5 M NaCl and 50 μg/mL GlycoBlue Coprecipitant (Thermo Fisher, USA) at −80 °C overnight. The precipitates of RNA were centrifuged at 14,000 rpm for 30 minutes at 4 °C, resuspended in 10 μL RNase-free water, and further detected by qRT-PCR.

### RNA fluorescent in situ hybridization (RNA-FISH)

DIG-labeled LUCAT1 probes were obtained from Servicebio (China), and hybridizations were carried out using their FISH Kit according to the manufacturer’s instructions. Briefly, cells were fixed with 4% paraformaldehyde, treated with 0.5% Triton, and incubated with a specific probe overnight. All fluorescence images were captured using a Nikon A1Si Laser Scanning Confocal Microscope (Nikon Instruments Inc., Japan). The sequence for LUCAT1 probe is: 5′-TCTCTTTATTTACAATACCTTTTCA-3′.

### Nucleocytoplasmic separation

Nuclear and cytosolic fractions were separated using the PARIS kit (Thermo Fisher Scientific, USA) as directed by the manufacturer. Then, the expression levels of GAPDH, U6, and LUCAT1 in the cytoplasm or nucleus of LUAD cells were detected using a qRT-PCR assay.

### Immunohistochemistry (IHC) analysis

Paraffin-embedded sections of tumor tissues from NCG mice were incubated at 60 °C for 2 h, followed by immersion. The sections were then rehydrated using different concentrations of ethanol (including 100%, 95%, 85%, 70%) and deionized water. Subsequently, these slices were immersed in a citrate buffer solution (0.01 mol/L, pH 6.0) and heated to maintain a temperature between 95 °C and 100 °C for 30 min. Following this, the sections were washed with PBS and incubated with 0.5% Triton X-100 for 30 min. Finally, the sections were stained using the biotin-streptavidin HRP detection system (ZSGB, China), and these slices were incubated with an anti-Ki67 primary antibody (1:200, #34330, CST) overnight at 4 °C. Positive immunoreactivity was observed as a brown chromogen in the membrane.

### TUNEL assay

The TUNEL assay was conducted utilizing the In Situ Cell Death Detection Kit (Roche, Germany). In accordance with the manufacturer’s instructions, tissue sections were deparaffinized and rehydrated. Antigen retrieval was achieved by employing a heated 0.1 M citrate buffer (pH 6.0), followed by incubation with the TUNEL reaction mixture, which comprised terminal deoxynucleotidyl transferase (TdT) and fluorescein-conjugated deoxyuridine triphosphate (dUTP), for 1 h at 37 °C. Nuclear staining was performed using DAPI. Apoptotic cells were subsequently analyzed via fluorescence microscopy (Leica, Germany).

### GW4869 and RNase A treatment

To inhibit the release of exosomes, LUAD cells subjected to hypoxic conditions were pretreated with GW4869 (an inhibitor of exosome formation and release, Sigma, USA) at a concentration of 10 μM for 24 h. For the RNase A treatment, exosomes were incubated with RNase A (Takara, Japan) at a final concentration of 10 μg/mL, either alone or in combination with 0.1% Triton X-100, for 20 min.

### Co-culture assay

The co-culture assay was conducted to investigate the ability of extracted exosomes to penetrate LUAD cells. Following revival and passaging, LUAD cells were seeded into a confocal culture dish containing RPMI-1640 medium and allowed to adhere completely. Concurrently, exosome extracts labeled with the green fluorescent dye PKH67 (Umibio Co., Ltd., China) were introduced for co-culture with the LUAD cells. After a 24-h incubation period, the culture was washed three times with PBS and subsequently examined and documented through micrographs using a confocal microscope (Zeiss LSM780, Germany).

### Western blot assay

Western blot assay was performed as previously described with slight modification [23, 24]. Briefly, proteins were extracted using RIPA buffer (Beyotime, China) supplemented with 1% phosphatase and protease inhibitor cocktails (Sigma, USA). Protein concentrations were then quantified using the BCA Protein Assay Kit (Transgene, China) following the manufacturer’s instructions. Equal amounts of each sample were separated by 10% SDS-PAGE and subsequently transferred onto NC membrane (PALL Corporation, USA). The membranes were then blocked with 5% skim milk for 1 h at room temperature, followed by incubation with primary antibodies at 4 °C overnight. Then, the membranes were incubated with the appropriate secondary antibody at 37 °C for 1 h. After washing with TBST, the blots were visualized using the ChemiDoc MP Imaging System (BIO-RAD, USA). All experiments were conducted at least in duplicate. All primary antibodies are listed in Supplementary Table S5.

### Bioinformatics analysis

The expression profiles of LUCAT1 and c-MET, as archived in The Cancer Genome Atlas (TCGA, https://portal.gdc.cancer.gov/) and the Gene Expression Omnibus (GEO, http://www.ncbi.nlm.nih.gov/geo/), were retrieved and subjected to analysis utilizing the ggplot2 package (version 3.3.3) within the R statistical software environment (version 3.6.3). Subsequently, OS, disease-specific survival (DSS), and first progression were selected as prognostic parameters to examine the association between differential expression of LUCAT1 or c-MET and the prognosis of patients with NSCLC.

### Statistical analysis

All experiments were performed in triplicate, and statistical data were reported as mean ± standard deviation (SD). Comparisons between two groups were analyzed using the Student’s *t* test. while one-way analysis of variance (ANOVA) was employed for comparisons involving more than two groups. The Fisher's exact test was performed to evaluate differences in proportions among various groups. Statistical analyses were executed using GraphPad Prism software (version 8.02, USA). For quantitative Western blot analysis, band intensities were normalized to GAPDH using ImageJ software (v1.53a, NIH). Dose–response curves for drug treatments were fitted with nonlinear regression using GraphPad Prism software. A *P* value of less than 0.05 was considered indicative of statistical significance across all analyses.

## Results

### HExos contribute to osimertinib resistance in EGFR-mutant LUAD cells

**Fig. 1:**
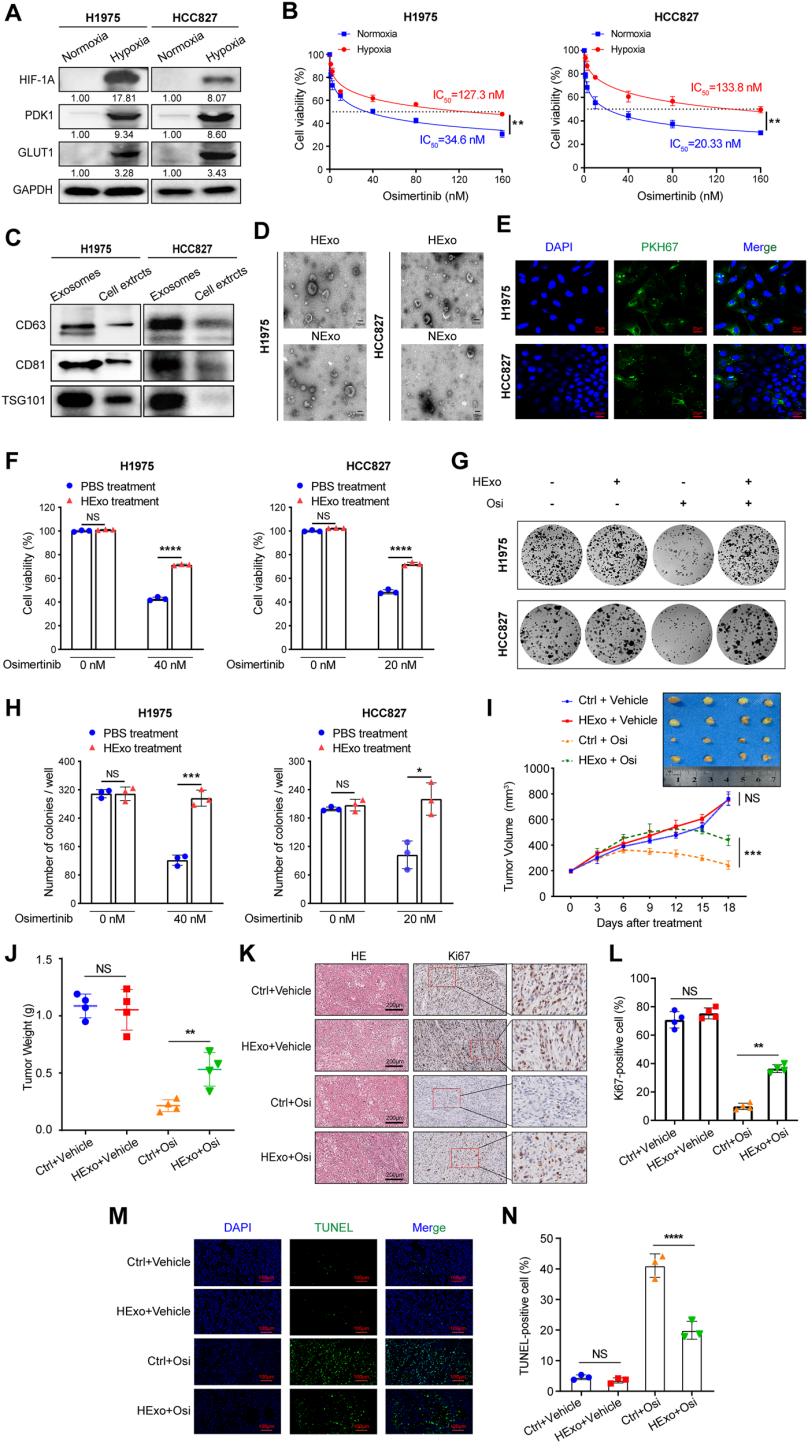
Hypoxia-derived exosomes induce EGFR-mutant LUAD osimertinib resistance in vitro and in vivo. **A** Immunoblots of HIF-1a, PDK1, GLUT1, and GAPDH (as control) in H1975 and HCC827 cells under hypoxia or normoxia. **B** Dose–response curves of hypoxic or normoxic H1975 and HCC827 cells treated for 48 h with osimertinib at the indicated concentrations. Data are represented as the mean ± SD; **P < 0.01, paired *t* test, *n* = 3. **C** Immunoblots of CD63, CD81, and TSG101 in exosomes derived from H1975 and HCC827 cells. **D** Representative transmission electron microscopy (TEM) images of osimertinib-resistant and parental (H1975 and HCC827) cells. Scale bar, 200 μm. **E** Immunofluorescence of H1975 and HCC827 cells incubation with PKH67-labeled (green) HExos. Scale bar, 20 μm. **F** Cell viability of H1975 and HCC827 cells pre-incubated with HExos (or PBS as control) for 24 h followed by osimertinib treatment at indicated concentrations for 48 h. Data are represented as the mean ± SD; ****P < 0.0001, and NS stands for no significance, paired *t* test, *n* = 3. **G**, **H** Colony-formation assays of H1975 (up) and HCC827 (bottom) cells co-cultured with HExos with osimertinib treatment at indicated concentrations for 2 weeks (*n* = 3). Average number of colonies (**H**) and representative images (**G**) are shown. Data are represented as the mean ± SD; *P < 0.05, ***P < 0.001 and NS stands for no significance, paired *t* test, *n* = 3. **I**, **J** Subcutaneous xenograft assay of H1975 cells (3 × 10^6^ cells) in NCG mice with vehicle or osimertinib (5 mg/kg) treatment. Volumes (**I**) and weight (**J**) of tumors are shown. Data are represented as the mean ± SD; **P < 0.01, ***P < 0.001, and NS stands for no significance, unpaired *t* test, *n* = 4. **K**, **L** H& E and IHC staining of Ki67 in xenograft tumors with vehicle or osimertinib (5 mg/kg) treatment. Quantification of IHC data for the positivity of Ki67 in the indicated groups was shown in (**L**). Scale bar, 200 μm. Data are represented as the mean ± SD. **P < 0.01 and NS stands for no significance, paired *t* test, *n* = 4. **M**, **N** TUNEL staining analysis of the proportion of apoptotic cells in xenograft tumors after incubation with vehicle or osimertinib (5 mg/kg). Quantification of TUNEL-positive cells in the indicated groups was shown in (**N**). Scale bar, 100 μm. Data are represented as the mean ± SD. ****P < 0.0001 and NS stands for no significance, paired *t* test, *n* = 3. TEM transmission electron microscopy, HExos hypoxic cells-derived exosomes, PBS phosphate-buffered saline, IHC immunohistochemistry.

It has been widely accepted that hypoxia as an important factor affects the malignant progression of solid tumors [25, 26]. In this study, based on experimental verification of protein expression levels for canonical hypoxia markers—HIF1α, GLUT1, and PDK1—we successfully established hypoxia models in LUAD cell lines H1975 and HCC827 (Fig. 1A). Subsequently, the sensitivity of LUAD cells to osimertinib under both normoxic and hypoxic conditions was investigated. The results of cell viability assays showed that the half maximal inhibitory concentration (IC_50_) for osimertinib was significantly higher in hypoxic LUAD cells compared to normoxic LUAD cells (Fig. 1B). Our earlier research indicated that HExos promote invasion, migration, and epithelial-to-mesenchymal transition processes in LUAD [12]. In addition, several studies have linked HExo to chemoresistance in various tumors [27, 28]. Based on these findings, we hypothesized that HExos secreted from LUAD cells might be a key mediator of osimertinib resistance. Therefore, we isolated and characterized exosomes from H1975 and HCC827 cell lines under normoxic and hypoxic conditions (Fig. 1C, D and Supplementary Fig. S1B). Subsequently, HExos from LUAD cells were co-incubated with normoxic H1975 and HCC827 cells, respectively (Supplementary Fig. S1C). Exosome uptake experiments revealed that HExo labeled with PKH67 could be internalized by neighboring LUAD cells in normoxic conditions (Fig. 1E). Furthermore, the results of CCK-8 and colony-formation assays consistently suggested HExos significantly promoted the resistance of LUAD cells to osimertinib (Fig. 1F–H). The role of HExos in promoting osimertinib resistance was further validated in a xenograft mouse model (Supplementary Fig. S1D). In NCG mice with subcutaneous tumors, HExos treatment induced osimertinib resistance specifically in the osimertinib-treated groups. Notably, in the absence of osimertinib pressure, HExos injection had no significant effects on tumor volume or weight (Fig. 1I, J). IHC and TUNEL assays of tumor tissues indicated that HExos inhibited osimertinib-induced apoptosis and enhanced cell proliferation (Fig. 1K–N), which further supported in vitro results.

### LUCAT1 is encapsulated and transferred by HExo and correlated with poor prognosis

**Fig. 2:**
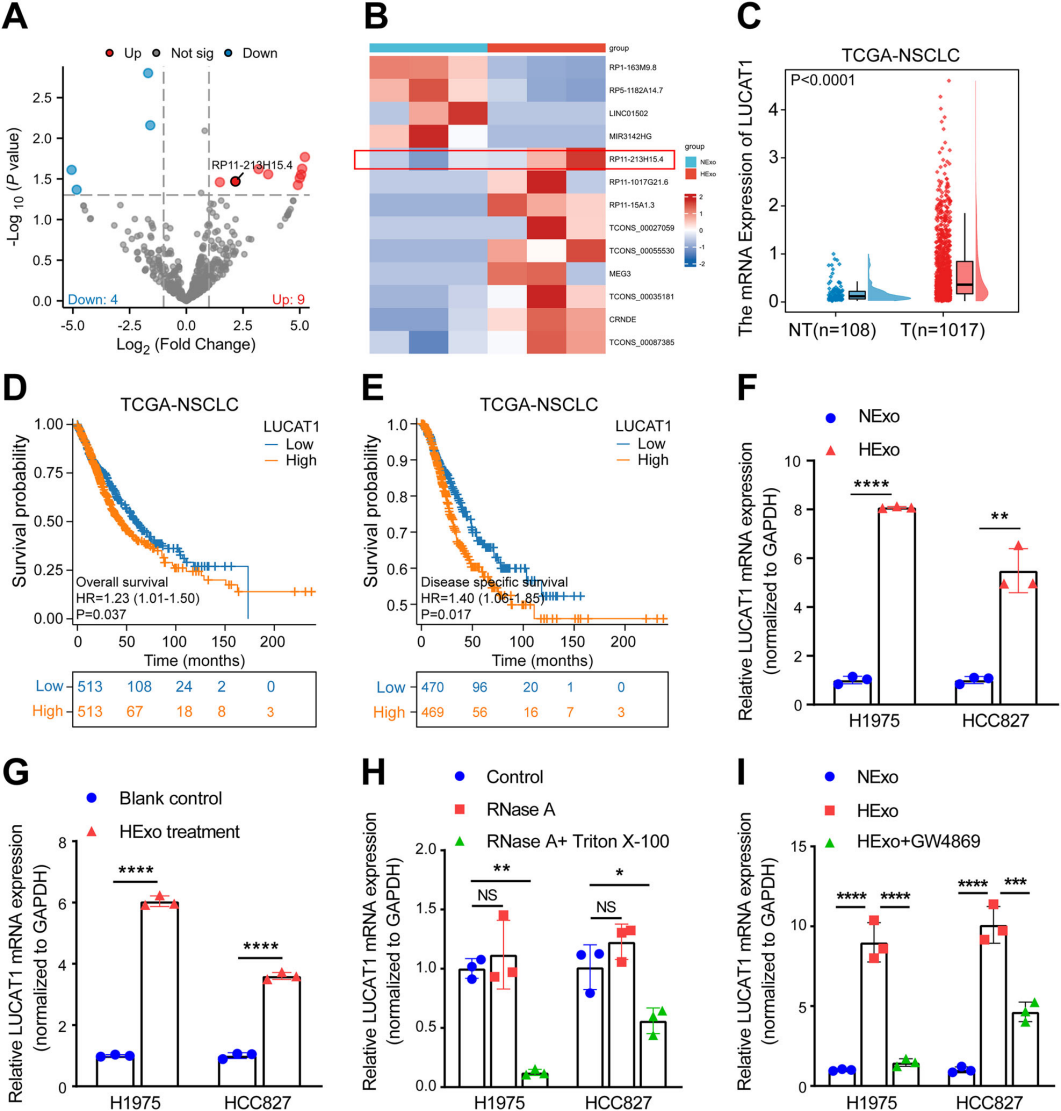
Identification of LUCAT1 in exosomes. **A**, **B** lncRNA sequence data of HExos and NExos derived from H1975 are presented in a volcano plot (**A**) and heatmap (**B**). **C** Relative expression level of LUCAT1 in adjacent para-tumor tissues (*n* = 108) and tumor tissues (*n* = 1017) from the TCGA cohort. Data are represented as the mean ± SD, ***P < 0.001, Wilcoxon rank-sum test. **D**, **E** Kaplan–Meier analysis of OS and DSS in the high and low LUCAT1 groups according to the median LUCAT1 level in NSCLC patients from the TCGA cohort. **F** qRT-PCR analysis of LUCAT1 in HExos and NExos derived from H1975 cells and HCC827 cells. Data are represented as the mean ± SD. **P < 0.01, ****P < 0.0001, paired *t* test, *n* = 3. **G** qRT-PCR analysis of LUCAT1 in H1975 and HCC827 cells 48 h after incubation with HExos (or PBS as control). Data are represented as the mean ± SD. ****P < 0.0001, paired *t* test, *n* = 3. **H** qRT-PCR analysis of LUCAT1 in the conditioned medium of H1975 and HCC827 cells treated with RNase A (2 mg/ml) alone or combined with Triton X-100 (0.1%). Data are represented as the mean ± SD. *P < 0.05, **P < 0.01, and NS stands for no significance, paired *t* test, *n* = 3. **I** qRT-PCR analysis of LUCAT1 in NExo, HExo, and HExo+GW4869 groups. Data are represented as the mean ± SD. ***P < 0.001 and ****P < 0.0001, paired *t* test, *n* = 3. HExos hypoxic cells-derived exosomes, NExos normoxic cells-derived exosomes, OS overall survival, DSS disease-specific survival, qRT-PCR quantitative real-time polymerase chain reaction.

To explore the underlying mechanism by which HExos promote osimertinib resistance in LUAD cells, RNA sequencing was performed to identify differentially expressed genes between HExos and NExos after depleting rRNA. Using a threshold of fold change (FC) = 2 and *P* < 0.05, we identified 13 differentially expressed lncRNAs (Fig. 2A, B and Supplementary Table S1). Among the nine lncRNAs with higher expression in the HExo group, lncRNA RP11-213H15.4 (Ensembl Transcript ID: ENST00000607854), also known as LUCAT1 (www.lncipedia.org), stood out due to its significant association with chemoresistance in NSCLC, colon cancer, and osteosarcoma [20, 21]. Subsequently, we investigated the role of LUCAT1 in NSCLC and found that LUCAT1 is highly expressed in NSCLC patients based on the TCGA database (Fig. 2C). In addition, Kaplan–Meier curves demonstrate that high LUCAT1 expression is significantly associated with poorer OS and DSS in NSCLC patients (Fig. 2D, E).

In addition, the qRT-PCR assay reveals that LUCAT1 is significantly upregulated in HExo from LUAD cells compared to NExo (Fig. 2F). Notably, after 24 h of co-culture with HExos, LUCAT1 expression was significantly elevated in the co-culture group compared to the control group (Fig. 2G). To verify that LUCAT1 is encapsulated within exosomes, we treated the samples with RNase A, which degrades free RNA, and Triton X-100, which disrupts the cell membrane. The expression levels of LUCAT1 remained unchanged in the RNase A treatment groups, but significantly decreased when both RNase A and Triton X-100 were applied (Fig. 2H). These results suggest that LUCAT1 is indeed transferred to other cells via LUAD cell-derived exosomes, and it is protected from degradation by the exosomal lipid bilayer. We treated cells under hypoxic conditions with GW4869 (an inhibitor of exosome synthesis and release) and found that, compared to the hypoxic group, the exosome content in the cell supernatant was significantly reduced in the hypoxia+GW4869 group (Supplementary Fig. S2A). Furthermore, exosomes isolated after GW4869 treatment could effectively reverse the high levels of LUCAT1 observed in HExo (Fig. 2I).

### LUCAT1 confers osimertinib resistance in EGFR-mutant LUAD cells

**Fig. 3:**
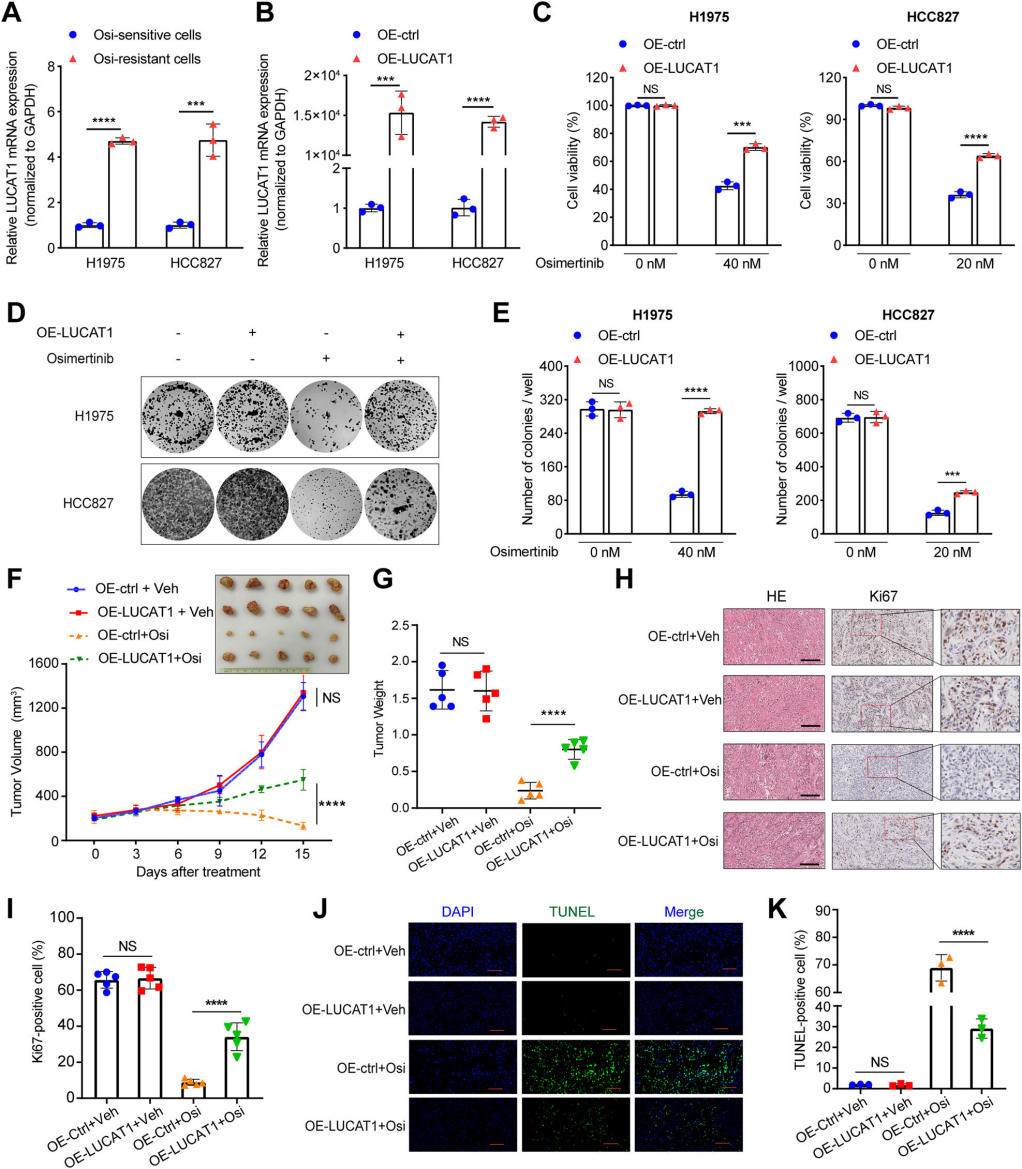
The upregulation of LUCAT1 promotes LUAD osimertinib resistance in vitro and in vivo. **A** qRT-PCR analysis of LUCAT1 in osimertinib-resistant and parental (H1975 and HCC827) cells. Data are represented as the mean ± SD. ***P < 0.001 and ****P < 0.0001, paired *t* test, *n* = 3. **B** qRT-PCR analysis of LUCAT1 in LUCAT1-overexpressing and control LUAD cells. Data are represented as the mean ± SD. ***P < 0.001 and ****P < 0.0001, paired *t* test, *n* = 3. **C** Cell viability of LUCAT1-overexpressing and control LUAD cells with osimertinib treatment at indicated concentrations for 48 h. Data are represented as the mean ± SD. ***P < 0.001, ****P < 0.0001, and NS stands for no significance, paired *t* test, *n* = 3. **D**, **E** Colony-formation assay of LUCAT1-overexpressing and control LUAD cells with osimertinib treatment at indicated concentrations in a six-well dish (1 × 10^3^ cells per well) for 2 weeks. Average number of colonies (**E**) and representative images (**D**) are shown. Data are represented as the mean ± SD. ***P < 0.001 and ****P < 0.0001, paired *t* test, *n* = 3. **F**, **G** Subcutaneous xenograft assay of LUCAT1-overexpressing and control H1975 cells (3 × 10^6^ cells) in NCG mice with vehicle or osimertinib (5 mg/kg) treatment. Volumes (**F**) and weight (**G**) of tumors are shown. Data are represented as the mean ± SD. ****P < 0.0001 and NS stands for no significance, unpaired *t* test, *n* = 5. **H**, **I** H& E and IHC staining of Ki67 in xenograft tumors with vehicle or osimertinib (5 mg/kg) treatment. Quantification of IHC data for the positivity of Ki67 in the indicated groups was shown in (**I**). Scale bar, 200 μm. Data are represented as the mean ± SD. ****P < 0.0001 and NS stands for no significance, paired *t* test, *n* = 5. **J**, **K** TUNEL staining analysis of the proportion of apoptotic cells in xenograft tumors after incubation with vehicle or osimertinib (5 mg/kg). Quantification of TUNEL-positive cells in the indicated groups was shown in (**K**). Scale bar, 100 μm. Data are represented as the mean ± SD. ****P < 0.0001 and NS stands for no significance, paired *t* test, *n* = 3. qRT-PCR quantitative real-time polymerase chain reaction, LUAD lung adenocarcinoma, IHC immunohistochemistry.

Next, we established OR LUAD cell lines (H1975/OR and HCC827/OR) by a stepwise dose-escalation method (Supplementary Fig. S3A–E). qRT-PCR assays revealed that the expression of LUCAT1 in the resistant cells was significantly higher compared to their parental counterparts (Fig. 3A). Afterward, we generated stable H1975 and HCC827 cells overexpressing LUCAT1 (Fig. 3B). CCK-8 assays and colony-formation assays demonstrated that overexpression of LUCAT1 did not enhance LUAD cell proliferation in the absence of osimertinib pressure. However, treatment with osimertinib, cells overexpressing LUCAT1 exhibited a significantly higher survival rate than the control cells (Fig. 3C–E). In vivo, tumor xenograft experiments confirmed that overexpression of LUCAT1 conferred resistance to osimertinib (Supplementary Fig. S3F and Fig. 3F, G). Moreover, the results of IHC indicated that Ki67 expression increased in the LUCAT1-overexpressing tumor tissues compared to the control group following osimertinib treatment, while no significant difference was observed between the two groups without the drug (Fig. 3H, I). TUNEL assays further confirmed that LUCAT1-overexpression could diminish cell apoptosis induced by osimertinib (Fig. 3J, K). These results strongly suggest that upregulation of LUCAT1 contributes to osimertinib resistance in EGFR-mutant LUAD.

**Fig. 4:**
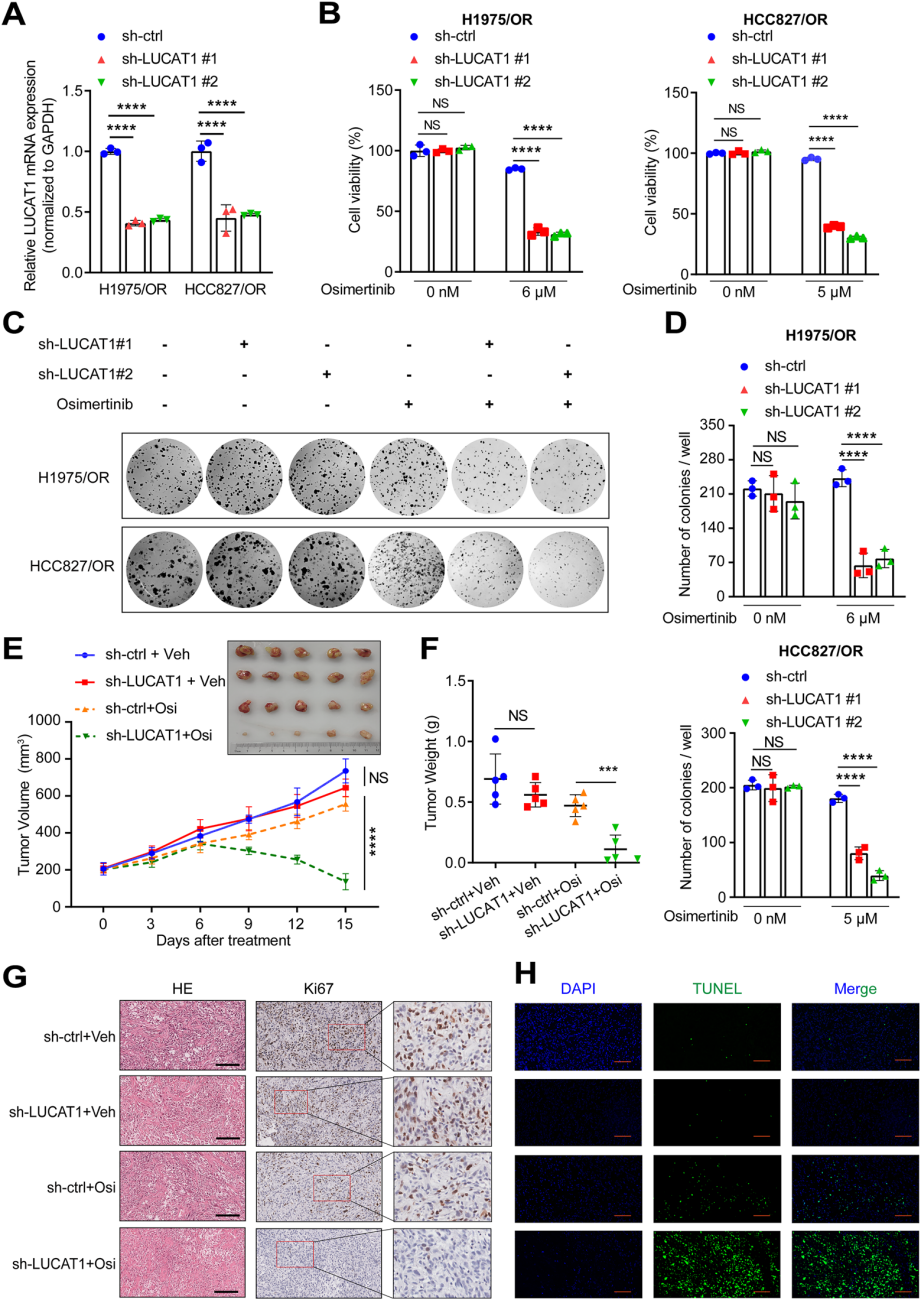
The knockdown of LUCAT1 restores the sensitivity of LUAD to osimertinib in vitro and in vivo. **A** qRT-PCR analysis of LUCAT1 in H1975/OR and HCC827/OR cells transfected with LUCAT1 shRNA after 72 h. Data are represented as the mean ± SD. ****P < 0.0001, paired *t* test, *n* = 3. **B** Cell viability of LUCAT1-knockdown and control H1975/OR (Left) and HCC827/OR (right) cells with osimertinib treatment at indicated concentrations for 48 h. Data are represented as the mean ± SD. ****P < 0.0001 and NS stands for no significance, paired *t* test, *n* = 3. **C**, **D** Colony-formation assay of LUCAT1-knockdown and control H1975/OR (up) and HCC827/OR (bottom) cells with osimertinib treatment at indicated concentrations in a six-well dish (1 × 10^3^ cells per well) for 2 weeks. Average number of colonies (**D**) and representative images (**C**) are shown. Data are represented as the mean ± SD. ****P < 0.0001 and NS stands for no significance, paired *t* test, *n* = 3. **E**, **F** Subcutaneous xenograft assay of LUCAT1-knockdown and control H1975/OR cells (3 × 10^6^ cells) in NCG mice with vehicle or osimertinib (5 mg/kg) treatment. Volumes (**E**) and weight (**F**) of tumors are shown. Data are represented as the mean ± SD. ***P < 0.001, ****P < 0.0001, and NS stands for no significance, unpaired *t* test, *n* = 5. **G** H& E and IHC staining of Ki67 in xenograft tumors with vehicle or osimertinib (5 mg/kg) treatment. Scale bar, 200 μm. **H** TUNEL staining analysis of the proportion of apoptotic cells in xenograft tumors after incubation with vehicle or osimertinib (5 mg/kg). Scale bar, 100 μm. qRT-PCR quantitative real-time polymerase chain reaction, OR osimertinib resistance, IHC immunohistochemistry.

To further validate the findings above, we stably knocked down LUCAT1 expression in OR cells (Fig. 4A). We found that knocked down LUCAT1 restored the sensitivity of these cells to osimertinib in vitro (Fig. 4B–D), and in vivo experiments confirmed that reducing LUCAT1 levels could reverse osimertinib resistance (Supplementary Fig. S4A and Fig. 4E, F). IHC and TUNEL assays showed that LUCAT1 knockdown reduced cell proliferation and promoted apoptosis in resistant cells (Fig. 4G, H and Supplementary Fig. S4B, C).

### Intercellular transfer of LUCAT1 by exosomes disseminates osimertinib resistance

**Fig. 5:**
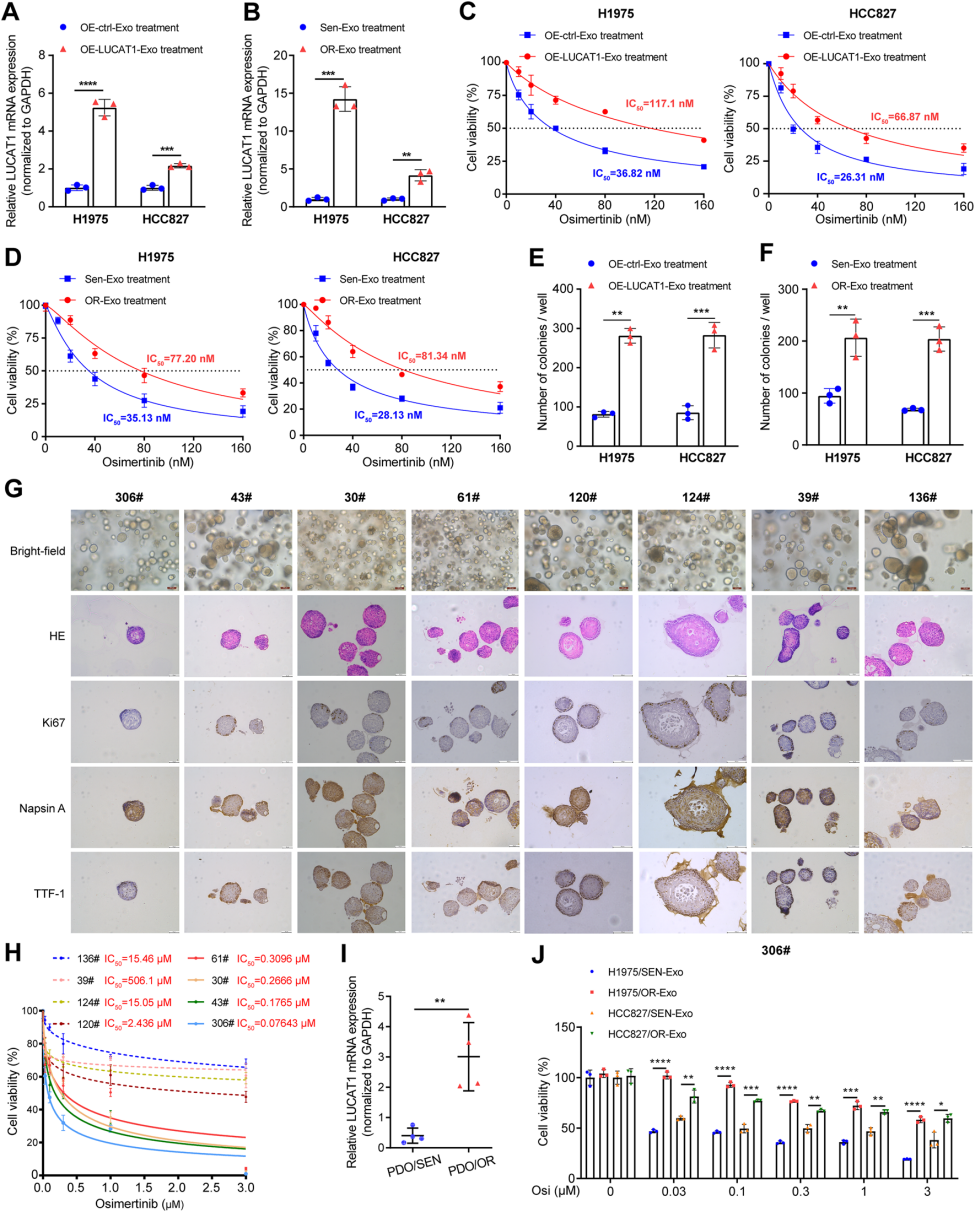
Intercellular transfer of LUCAT1 by resistant cells-derived exosomes disseminates osimertinib resistance. **A** qRT-PCR analysis of LUCAT1 in H1975 and HCC827 cells incubated with exosomes derived from LUCAT1-overexpressing (OE) LUAD cells for 48 h. Data are represented as the mean ± SD. ***P < 0.001 and ****P < 0.0001, paired *t* test, *n* = 3. **B** qRT-PCR analysis of LUCAT1 in H1975 and HCC827 cells incubated with exosomes derived from osimertinib-resistant (OR) LUAD cells for 48 h. Data are represented as the mean ± SD. **P < 0.01 and ***P < 0.001, paired *t* test, *n* = 3. **C**, **D** Cell viability of H1975 and HCC827 cells incubated with indicated exosomes with osimertinib treatment at indicated concentrations for 48 h (*n* = 3). **E**, **F** Colony-formation assays of H1975 and HCC827 cells incubated with indicated exosomes with osimertinib treatment at indicated concentrations for 3 weeks. Data are represented as the mean ± SD. **P < 0.001 and ***P < 0.001, paired *t* test, *n* = 3. **G** Representative bright-field, H&E staining, and IHC staining of Ki67, Napsin A, and TTF-1 in tumor organoids originating from eight EGFR-mutant LUAD patients. **H** Organoid viability ATP assay of EGFR-mutant LUAD organoids to osimertinib at indicated concentrations for 3 days (*n* = 3). **I** qRT-PCR analysis of LUCAT1 in the EGFR-mutant LUAD organoids. Data are represented as the mean ± SD. **P < 0.001, paired *t* test, *n* = 3. **J** Organoid viability ATP assay of 306# organoid incubated with indicated exosomes (40 μg/ml) with osimertinib treatment at indicated concentrations for 3 days. Data are represented as the mean ± SD. *P < 0.05, **P < 0.01, ***P < 0.001 and ****P < 0.0001, paired *t* test, *n* = 3. LUAD lung adenocarcinoma, OE overexpression, OR osimertinib resistant.

To explore the effects of exosomes from different sources on osimertinib resistance in EGFR-mutant LUAD cells, we extracted exosomes from stable LUCAT1-overexpressing cells and OR cells, respectively. As anticipated, the intercellular levels of LUCAT1 were significantly elevated following incubation with exosomes from LUCAT1-overexpressing cells and OR cells (Fig. 5A, B). In addition, parental LUAD cells incubated with these exosomes exhibited decreased sensitivity to osimertinib (Fig. 5C–F and Suppplementary Fig. S5A, B). Organoids provide a unique benefit by developing in 3D, often mimicking the natural structure of the tissue they originate from. This allows them to better replicate the in vivo tumor environment compared to traditional 2D cultures on plastic [29]. Therefore, to further confirm that exosomes from resistant cells can disseminate resistance to other cells, we constructed eight LUAD organoids derived from EGFR-mutant LUAD patients (Fig. 5G). Drug sensitivity testing revealed that four of these LUAD organoids (136#, 19#, 124#, 120#) were resistant to osimertinib treatment, while the remaining four samples (61#, 30#, 43#, 306#) were sensitive (Fig. 5H). We extracted RNA from these organoids, which showed significantly higher LUCAT1 expression in the relatively resistant organoids compared to the sensitive ones (Fig. 5I). We then co-cultured 306# organoid sample with exosomes derived from both parental cells and resistant cells (Supplementary Fig. S5C). The results of the drug sensitivity assay indicated that exosomes secreted from OR cells significantly decreased the sensitivity of the EGFR-mutant LUAD organoids to osimertinib (Fig. 5J). Collectively, these findings demonstrated that exosomes from resistant cells can confer osimertinib resistance to recipient LUAD cells through the intercellular transfer of LUCAT1, thereby disseminating resistance to osimertinib.

### LUCAT1 upregulates the protein levels of c-MET by preventing from proteasome-dependent degradation

**Fig. 6:**
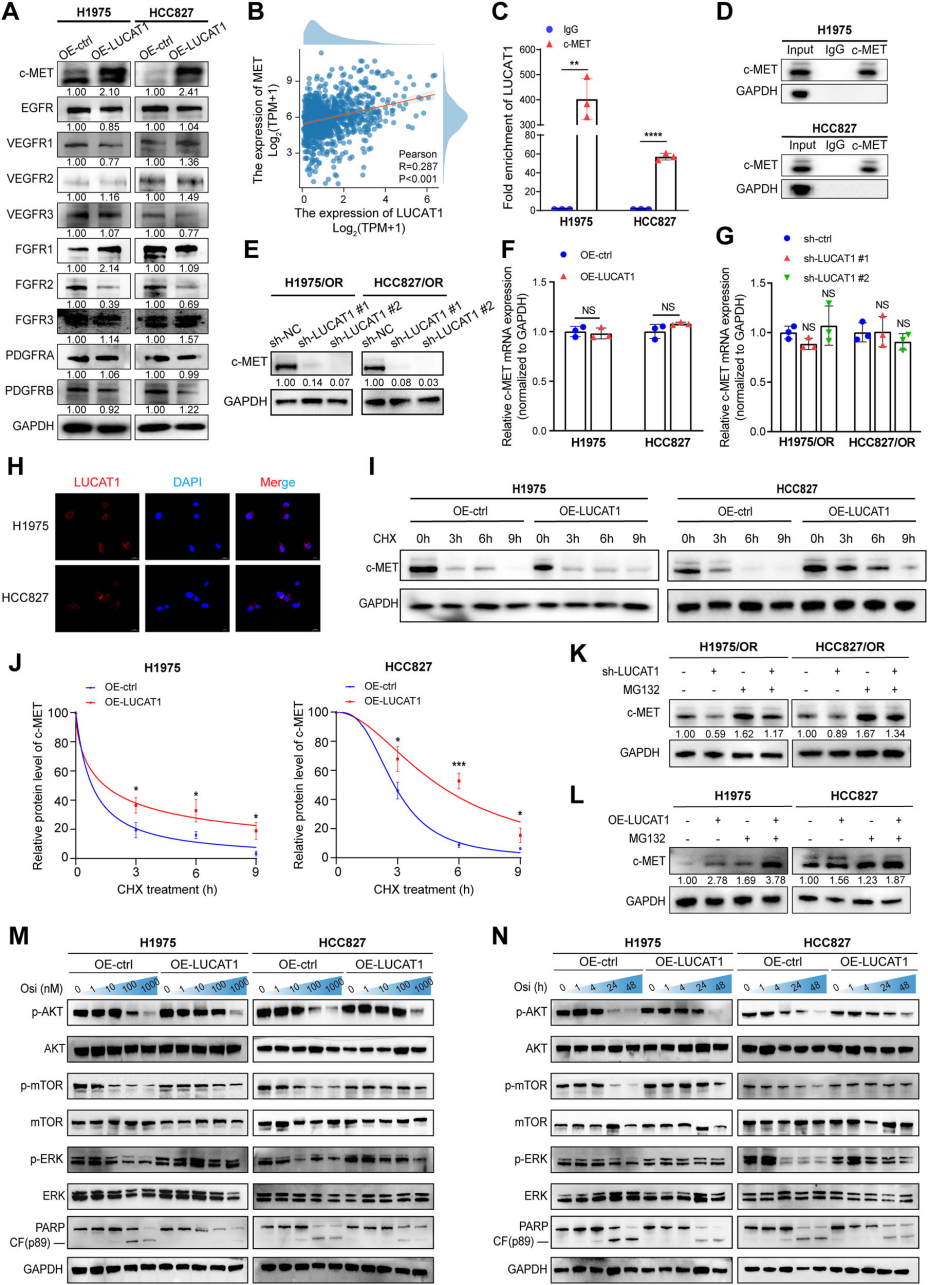
LUCAT1 upregulated c-MET protein expression by preventing its degradation. **A** Immunoblots of indicated proteins in LUCAT1-overexpressing and control H1975 and HCC827 cells. **B** Pearson correlation analysis between LUCAT1 levels and c-MET in NSCLC tissues (*n* = 1041) from TCGA cohort. **C**, **D** RIP assays of the interaction of c-MET and LUCAT1 in H1975 and HCC827 cells. Data are represented as the mean ± SD. **P < 0.01 and ***P < 0.001, paired *t* test, *n* = 3. **E** Immunoblots of c-MET in LUCAT1-knockdown and control H1975/OR and HCC827/OR cells. **F**, **G** qRT-PCR analysis of c-MET in LUCAT1-overexpressing and control H1975 and HCC827 cells (**I**) and in LUCAT1-knockdown and control H1975/OR and HCC827/OR cells (**J**). Data are represented as the mean ± SD. NS stands for no significance, paired *t* test, *n* = 3. **H** Fluorescence in situ hybridization analysis of LUCAT1 in H1975 and HCC827 cells. The nuclei were stained with DAPI. Scale bar, 20 μm. **I**, **J** Immunoblots of stability of c-MET protein in LUCAT1-overexpressing and control H1975 and HCC827 cells treated with 50 μg/ml cycloheximide (CHX) at the indicated intervals. Data are represented as the mean ± SD. *P < 0.05 and ***P < 0.001, paired *t* test, *n* = 3. **K**, **L** Immunoblots of c-MET protein in LUCAT1-overexpressing and control H1975 and HCC827 cells (up) and LUCAT1-knockdown and control H1975/OR and HCC827/OR cells (bottom) treated with MG132 (30 μM, 3 h). **M** Immunoblots of indicated proteins in LUCAT1-overexpressing and control H1975 and HCC827 cells with osimertinib treatment at indicated concentrations for 24 h. **N** Immunoblots of indicated proteins in LUCAT1-overexpressing and control H1975 and HCC827 cells with osimertinib treatment (100 nM) for the indicated times. NSCLC non-small cell lung cancer, TCGA The Cancer Genome Atlas, RIP RNA binding protein immunoprecipitation, qRT-PCR quantitative real-time polymerase chain reaction, OR osimertinib resistant.

Accumulating evidence highlighted the activation of alternative receptor tyrosine kinases (RTKs) as critical contributors to drug resistance [30, 31]. Therefore, we investigated the expression of ten RTKs, including c-MET, EGFR, VEGFRs, FGFRs, and PDGFRs, in LUCAT1-overexpressing cells and LUCAT1-control cells. Western blot results showed a significant upregulation in c-MET protein levels but not other RTKs (Fig. 6A). Bioinformatics analysis and qRT-PCR further showed that c-MET is upregulated in NSCLC and strongly associated with poor prognosis (Supplementary Fig. S6A–E). In addition, a significant positive correlation was identified between LUCAT1 and c-MET gene expression in the TCGA database (*R* = 0.287, *P* < 0.001, Fig. 6B). RIP-qPCR assays confirmed that there was an interaction between LUCAT1 and c-MET (Fig. 6C, D). Interestingly, as the expression of LUCAT1 changes, the protein levels of c-MET also vary accordingly, while the mRNA levels of c-MET did not show a significant alteration (Fig. 6E–G). These findings suggested that LUCAT1 may modulate c-MET at the post-transcriptional level. FISH and nuclear-cytoplasmic fractionation experiments indicated that LUCAT1 predominantly localized in the cytoplasm, which further supported our results described above (Fig. 6H and Supplementary Fig. S6F). Subsequently, we performed a CHX chase assay and found that the degradation rate of c-MET was markedly slower in the LUCAT1-overexpressing cells group compared to the control group (Fig. 6I, J). In addition, treatment with MG132, the proteasome inhibitor, attenuates the regulatory effect of LUCAT1 on c-MET protein level (Fig. 6K, L), further supporting the idea that LUCAT1 may stabilize c-MET by inhibiting its proteasomal degradation to induce osimertinib resistance in EGFR-mutant LUAD cells.

Previous study has discovered that the aberrant activation of the PI3K/AKT/mTOR and MAPK signaling pathways contributes to tumor cell resistance [32, 33]. In this study, we observed that osimertinib treatment at different concentrations inhibited the AKT/mTOR and ERK signaling pathways. Specifically, treatment with higher concentrations of osimertinib (over 100 nM), resulted in that the phosphorylation levels of AKT, mTOR, and ERK were significantly decreased in the control group. However, this inhibitory effect was markedly reduced in the LUCAT1-overexpressing group (Fig. 6M, N). In addition, cleaved-PARP protein expression was significantly lower in LUCAT1-overexpressing LUAD cells compared to the control group. When treated with 100 nM osimertinib for various durations, significant differences in the phosphorylation levels of AKT, mTOR, and ERK were observed between the LUCAT1-overexpressing and control cells. Collectively, these results suggest that LUCAT1 may induce osimertinib resistance in LUAD by stabilizing c-MET and activating AKT/mTOR and ERK signaling pathways.

### c-MET mediates LUCAT1-induced osimertinib resistance of LUAD cells

**Fig. 7:**
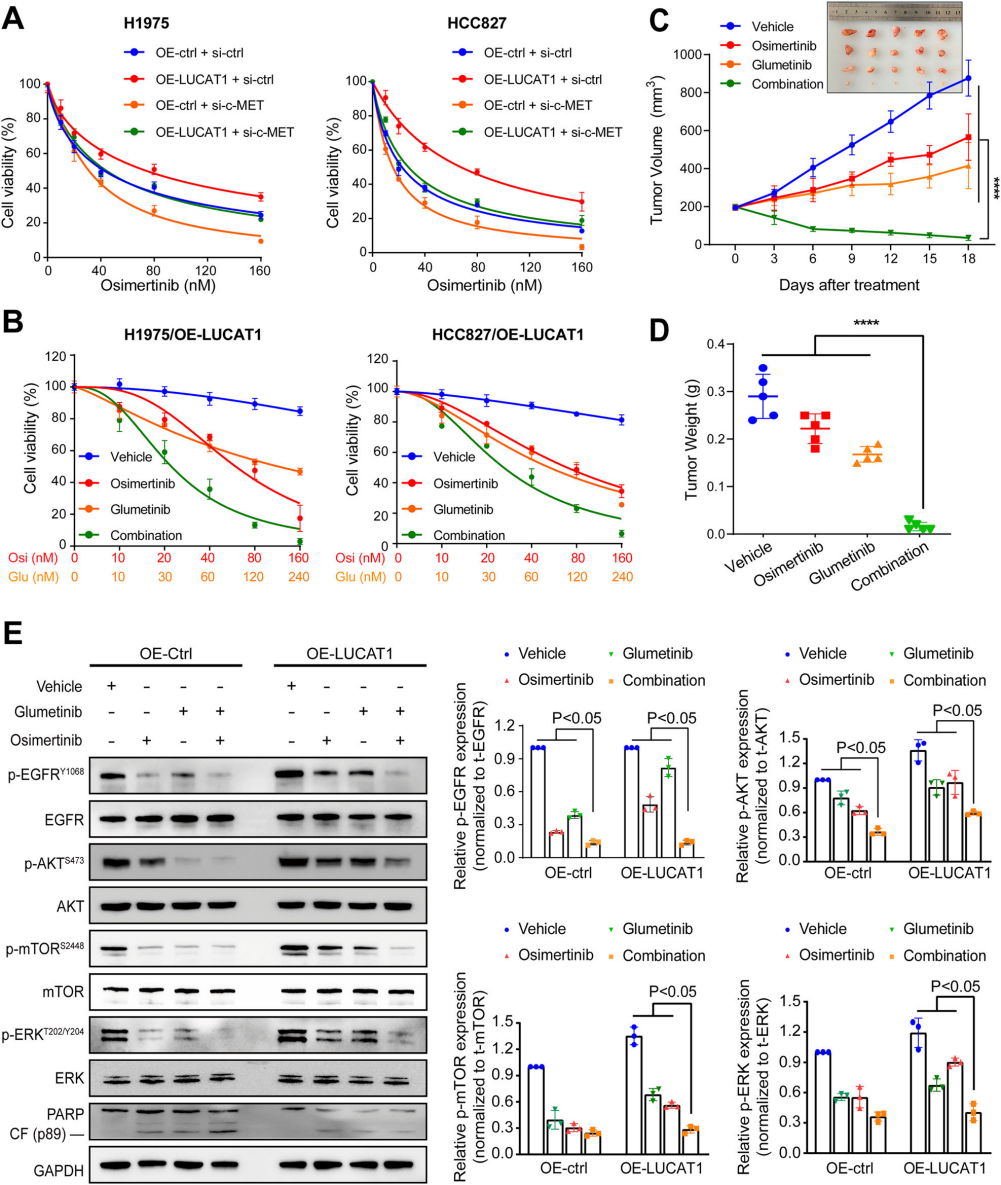
c-MET mediated the function of LUCAT1 in osimertinib resistance. **A** Dose–response curves of H1975 and HCC827 cells transfected with the indicated vector upon osimertinib treatment at the indicated concentrations for 48 h. **B** Dose–response curves of LUCAT1-overexpressing H1975 and HCC827 cells with osimertinib and glumetinib treatment alone or in combination at indicated concentrations for 48 h. **C**, **D** Subcutaneous xenograft assay of LUCAT1-overexpressing and control H1975 cells (3 × 10^6^ cells) in NCG mice treated with vehicle or osimertinib (5 mg/kg) along with glumetinib (5 mg/kg). Volumes (**C**) and weight (**D**) of tumors are shown. Data are represented as the mean ± SD. ****P < 0.0001, one-way ANOVA, *n* = 5. **E** Immunoblots of indicated proteins in LUCAT1-overexpressing and control H1975 cells treated with vehicle or osimertinib along with glumetinib for 24 h. The intensity of p-EGFR/p-AKT/p-mTOR/p-ERK were normalized to total EGFR/AKT/mTOR/ERK expression and quantified by ImageJ. Data are represented as the mean ± SD. *P* < 0.05 indicates statistical significance, one-way ANOVA, *n* = 3.

To confirm that LUCAT1-induced osimertinib resistance in EGFR-mutant LUAD cells is mediated by c-MET protein expression, we performed c-MET knockdown in stable LUCAT1-overexpressing cells (Supplementary Fig. S7A). The results of the cell viability assay demonstrated that c-MET knockdown significantly reversed the osimertinib resistance of LUAD cells with LUCAT1 overexpression compared to the control group (Fig. 7A). Moreover, western blot analysis demonstrated that c-MET knockdown effectively reversed the activation of p-AKT, p-mTOR, and p-ERK induced by LUCAT1 overexpression (Supplementary Fig. S7A), directly proving that LUCAT1 promotes osimertinib resistance through the c-MET/AKT/mTOR and ERK signaling pathways. In addition, glumetinib, a c-MET-specific inhibitor, was used to evaluate the role of c-MET in osimertinib resistance of LUCAT1-overexpressing LUAD cells. The results indicated that the combination of glumetinib and osimertinib suppressed tumor growth more strongly than the monotherapy in LUCAT1-induced resistant cells (Fig. 7B). In vivo, the combined treatment group showed significantly reduced tumor volume and weight compared to other control groups (Supplementary Fig. S7B and Fig. 7C, D). Western blot results further supported these findings, revealed that the combined treatment significantly blocked the phosphorylation levels of AKT, mTOR, ERK, and enhanced the expression of cleaved-PARP in tumor tissues (Fig. 7E). These results collectively suggest that c-MET plays a pivotal role in LUCAT1-mediated osimertinib resistance and that targeting c-MET can effectively restore sensitivity to osimertinib in EGFR-mutant LUAD with high LUCAT1 expression.

### Plasma exosomal LUCAT1 as a diagnostic biomarker for osimertinib resistance

**Fig. 8:**
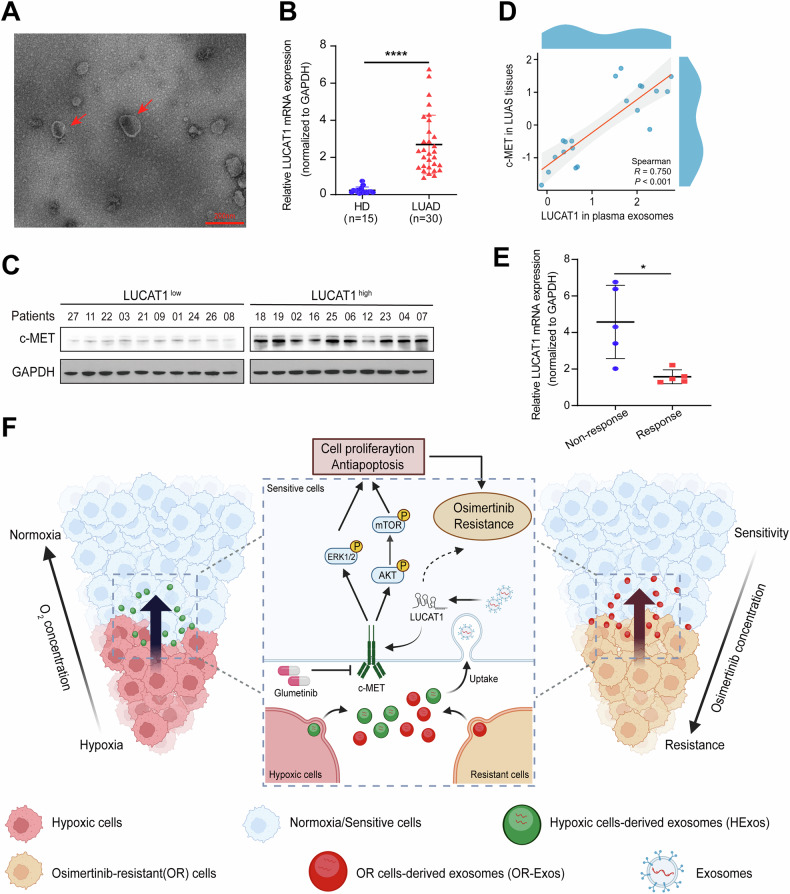
Exosomal LUCAT1 acts as a diagnostic biomarker for osimertinib resistance in EGFR-mutant LUAD. **A** A representative TEM image of plasma-derived exosomes from LUAD patients. **B** qRT-PCR analysis of LUCAT1 in LUAD patients (*n* = 30) and healthy donors (*n* = 15) plasma-derived exosomes. Data are represented as the mean ± SD. ****P < 0.0001, unpaired *t* test. **C** Immunoblots of c-MET and GAPDH in LUAD tissues. **D** Spearson correlation analysis between LUCAT1 levels in plasma-derived exosomes and c-MET levels in LUAD tissues (*n* = 20). **E** qRT-PCR analysis of LUCAT1 in the pre-therapy plasma-derived exosomes of EGFR-mutant LUAD patients responding (*n* = 5) or not responding (*n* = 5) to osimertinib therapy. Data are represented as the mean ± SD. *P < 0.05, unpaired *t* test. **F** A schematic diagram of exosomal LUCAT1 in LUAD osimertinib resistance. TEM transmission electron microscopy, LUAD lung adenocarcinoma, qRT-PCR quantitative real-time polymerase chain reaction.

Previous studies have highlighted a significant correlation between LUCAT1 and the malignant progression of various cancers [34, 35]. To further investigate whether plasma exosomal LUCAT1 could be a viable biomarker for osimertinib resistance in LUAD, we collected plasma samples from 30 LUAD patients and 15 HDs. Exosomes from the plasma were isolated and characterized (Fig. 8A). Subsequent RNA extraction from these plasma-derived exosomes revealed that exosomal LUCAT1 expression was significantly higher in LUAD patients compared to healthy donors (Fig. 8B). We further examined the relationship between LUCAT1 expression in plasma exosomes and c-MET protein levels in LUAD tissues. We analyzed the LUAD tissues of the top ten patients with the highest and lowest LUCAT1 levels. Our results showed that patients with higher LUCAT1 expression in plasma exosomes also had markedly elevated c-MET protein levels in their LUAD tissues (Fig. 8C). Spearman correlation analysis confirmed a strong positive correlation between LUCAT1 expression in plasma-derived exosomes and c-MET protein expression in tissue samples (*R* = 0.770, *P* < 0.001, Fig. 8D). Furthermore, we analyzed the plasma exosomal LUCAT1 levels in ten EGFR-mutant LUAD patients treated with osimertinib. Among these, five patients achieved a partial response defined by the Response Evaluation Criteria in Solid Tumors (RECIST; version 1.1) [36], while the remaining five had a poor response to osimertinib. Notably, the expression of LUCAT1 in plasma exosomes was significantly higher in the patients with poor response compared to those with a better treatment outcome (Fig. 8E). Collectively, these findings suggest that exosomal LUCAT1 might serve as a potential biomarker for the diagnosis of osimertinib resistance in LUAD.

## Discussion

Increasing evidences suggest that a hypoxic microenvironment is intricately associated with the malignant progression of solid tumors [37, 38]. Additionally, exosomes, extracellular vesicles with diameters of 30–150 nm, are emerging as important roles in carcinogenesis by reshaping the tumor microenvironment [39]. Previous studies have found that exosomes derived from hypoxic tumor cells can promote chemoresistance in tumors [14, 40]. In this study, we found that hypoxia significantly decreased the sensitivity of EGFR-mutant LUAD cells to osimertinib, highlight the complexity of the tumor microenvironment in drug resistance. Moreover, exosomes derived from EGFR-mutant LUAD cells under hypoxia condition could promote the resistance of LUAD cells to osimertinib, which strongly supports and extends the general concept of “hypoxic exosome-mediated drug resistance” to the specific context of osimertinib resistance in EGFR-mutant LUAD.

LUCAT1, a well-known lncRNA, has been identified to be upregulated in LUCA, breast cancer, and hepatocellular carcinoma, and participates in the regulation of proliferation, ferroptosis, migration, invasion, and even chemoresistance by sponging miRNAs [41–43]. However, it remains unclear whether LUCAT1 is involved in small-molecule drug resistance, particularly targeted drugs. In this study, our analysis demonstrated that LUCAT1 expression was significantly upregulated in NSCLC tissues, which correlated with worse overall survival in patients. More importantly, we have for the first time uncovered a novel biological function for LUCAT1, namely, exosomal LUCAT1 induces osimertinib resistance in EGFR-mutant LUAD cells. Our data demonstrated that LUCAT1 overexpression confers osimertinib resistance in EGFR-mutant LUAD cells, while its knockdown restores drug sensitivity, directly linking this lncRNA to the failure of first-line targeted therapy. Furthermore, we provide a novel mechanistic explanation: LUCAT1 was found to post-transcriptionally stabilizes c-MET, inhibiting its proteasome-dependent degradation and activates AKT/mTOR and ERK signaling pathways—a mechanism previously unreported in this setting. Meanwhile, our results also suggested that the expression of LUCAT1 in plasma exosomes is markedly positively correlated with the protein expression of c-MET in LUAD tissues of corresponding patients, and the levels of plasma exosomal LUCAT1 were significantly lower in patients who revealed good or partial responses than those who showed poor therapy responses. These findings further indicated that LUCAT1 may serve a critical role in inducing osimertinib resistance of EGFR-mutant LUAD cells. LUCAT1/c-MET axis might be a reliable biomarkers that predict the clinical benefits of osimertinib treatment. Notably, we demonstrated that combining osimertinib with the c-MET inhibitor (glumetinib) effectively reversed LUCAT1-driven resistance, validating the functional significance of this axis and suggesting a potential therapeutic strategy.

The concept that exosomes transfer functional molecules (including lncRNAs) to disseminate drug resistance phenotypes among cancer cells has been well-established [44]. Our study provides concrete evidence for this phenomenon in osimertinib resistance. We demonstrated that exosomes derived from either OR cells or LUCAT1-overexpressing cells can transfer LUCAT1 to recipient cells, effectively conferring osimertinib resistance upon them. This was validated not only in 2D cell cultures but also in the more physiologically relevant 3D LUAD organoid model. These findings further suggest that exosomes derived from drug-resistant cells may play a pivotal role in inducing osimertinib resistance in recipient cells by remodeling the tumor microenvironment.

While this study elucidated the critical role of exosomal LUCAT1 in mediating osimertinib resistance in EGFR-mutant LUAD cells and its potential as a diagnostic biomarker, several limitations warrant acknowledgment. First, the relatively small sample size (30 LUAD patients’ plasma exosomes, with only ten patients receiving osimertinib treatment and efficacy evaluation) may limit the generalizability of our findings. Future studies should validate the association between plasma exosomal LUCAT1 levels and osimertinib resistance/prognosis in larger, multicenter clinical cohorts. In addition, longitudinal studies are needed to assess its utility in dynamically monitoring resistance development. Second, while this study primarily focused on the LUCAT1/c-MET axis, tumor drug resistance is generally the result of multifactorial and multi-pathway interactions. Future research should further investigate whether LUCAT1 interacts with other known osimertinib resistance mechanisms (such as bypass activation and histological transformation), as well as its potential role in tumor microenvironment remodeling (e.g., immune suppression). Finally, although cell lines and organoid models effectively simulated key phenotypes, they cannot fully recapitulate the complex heterogeneity of in vivo tumors. Future studies should incorporate patient-derived xenograft models or advanced organoid co-culture systems to more authentically mimic exosome-mediated resistance propagation in the tumor microenvironment.

In conclusion, our findings uncovered that HExos or OR cells-derived exosomes could disseminate osimertinib resistance through intercellular transfer of LUCAT1. This also suggests that LUCAT1 in exosomes could be an important biomarker for osimertinib resistance in EGFR-mutant LUAD. On the other hand, we found that LUCAT1 induces osimertinib resistance in LUAD by inhibiting the degradation of c-MET and activating its downstream signaling pathways, which provides robust scientific evidence for targeting LUCAT1 or c-MET to overcome acquired resistance to osimertinib and potentially other third-generation EGFR-TKIs. Furthermore, plasma exosomal LUCAT1 might be a liquid biopsy target for evaluating patients for osimertinib treatment.

## Supplementary information

Supplementary Figures

Western Blot_Full unedited gel

Supplementary Table S1-S5

## Data Availability

All data related to this study have been included in the article and its supplementary information. RNA-seq data from The Cancer Genome Atlas (TCGA) analyzed in this study were obtained from the TCGA data portal at https://tcga-data.nci.nih.gov/tcga/. The microarray data between HExos and NExos in this study have been deposited in the National Genomics Data Center under the accession number PRJCA043958. Other data are available from the corresponding author on reasonable request.
