# Supplementary material for: Hypoxia-induced exosomal LUCAT1 promotes osimertinib resistance in lung adenocarcinoma by stabilizing c-MET
Source: Cell Death Dis. 2025 Oct 27;16(1):763. doi: 10.1038/s41419-025-08100-2 (PMC12559404; doi:10.1038/s41419-025-08100-2)
Supplement: Supplementary file 1 — Supplementary Figures [file 41419_2025_8100_MOESM1_ESM.pdf]

Supplementary Materials for

Hypoxia-induced Exosomal LUCAT1 Promotes Osimertinib Resistance in Lung Adenocarcinoma by Stabilizing c-MET

Jianting Du *et al.*

*Corresponding author. Email: chenchun0209@fjmu.edu.cn

This PDF file includes:

Figs. S1 to S7 and Supplementary legends

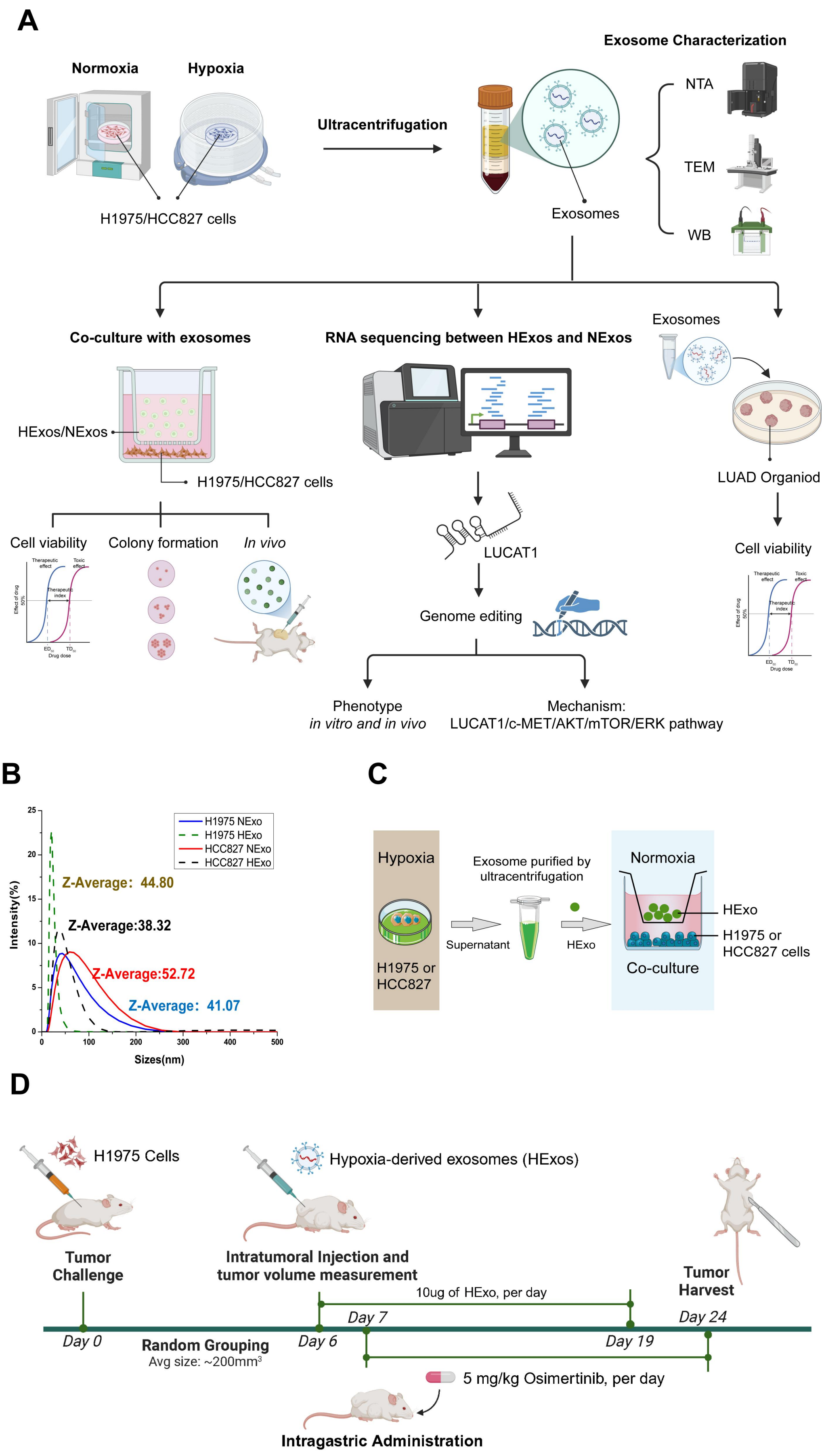
A
Normoxia
Hypoxia
H1975/HCC827 cells
Ultracentrifugation
Exosome Characterization
NTA
TEM
WB
Exosomes
Co-culture with exosomes
RNA sequencing between HExos and NExos
Exosomes
HExos/NExos
H1975/HCC827 cells
LUAD Organiod
Cell viability
Colony formation
In vivo
LUCAT1
Cell viability
Genome editing
Phenotype
in vitro and in vivo
Mechanism:
LUCAT1/c-MET/AKT/mTOR/ERK pathway
B
H1975 NExo
H1975 HExo
HCC827 NExo
HCC827 HExo
Z-Average: 44.80
Z-Average:38.32
Z-Average:52.72
Z-Average: 41.07
Intensity(%)
Sizes(nm)
C
Hypoxia
H1975 or HCC827
Exosome purified by ultracentrifugation
Supernatant
HExo
Normoxia
HExo
H1975 or HCC827 cells
Co-culture
D
H1975 Cells
Hypoxia-derived exosomes (HExos)
Tumor Challenge
Intratumoral Injection and tumor volume measurement
Tumor Harvest
10ug of HExo, per day
Day 0
Random Grouping
Avg size: ~200mm³
Day 6
Day 7
Day 19
Day 24
5 mg/kg Osimertinib, per day
Intragastric Administration

Supplementary Figure 1 related to Figure 1.

(A) A workflow of study design.

(B) Dynamic light scattering assays of the size of the indicated exosomes.

(C) Schematic model presenting the process to isolate HExo and co-cluture with LUAD cells.

(D) A schematic diagram of the experimental design used to establish the animal model.

NTA, Nanoparticle tracking analysis; TEM, transmission electron microscopy; WB, western blot; HExos, hypoxic cells-derived exosomes; NExos, normoxic cells-derived exosomes; LUAD, lung adenocarcinoma.

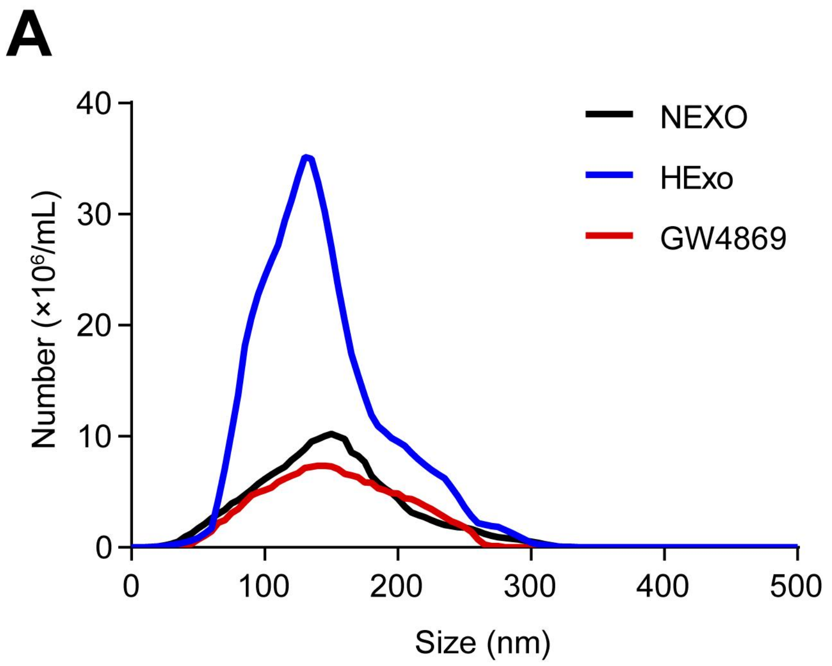

Supplementary Figure 2 related to Figure 2.

(A) Nanoparticle tracking analysis of the size distribution and number of exosomes.

NExo, normoxic cells-derived exosome; HExo, hypoxic cells-derived exosome.

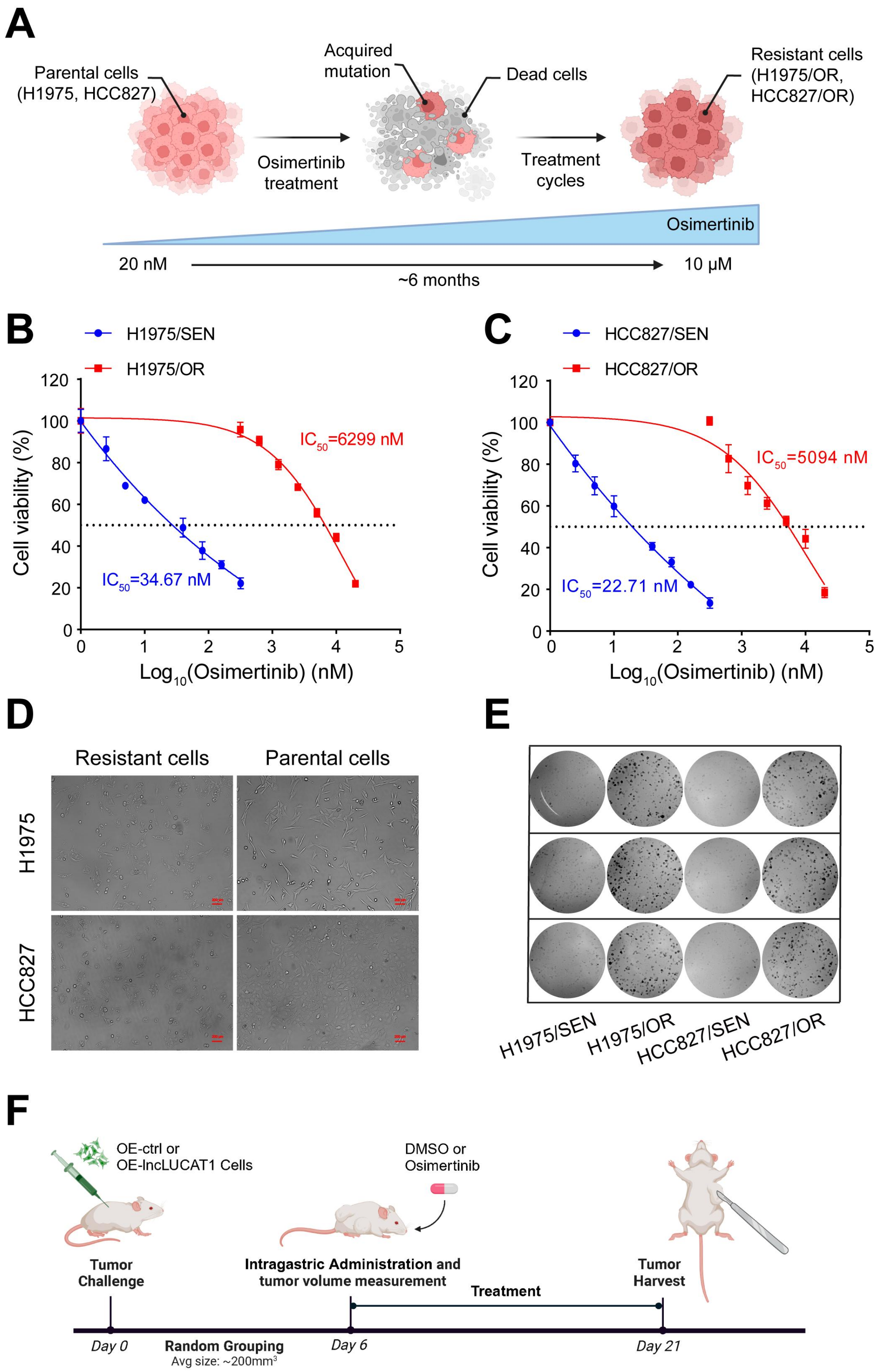
A
Parental cells
(H1975, HCC827)
Osimertinib
treatment
Acquired
mutation
Dead cells
Treatment
cycles
Resistant cells
(H1975/OR,
HCC827/OR)
Osimertinib
20 nM
~6 months
10 μM
B
H1975/SEN
H1975/OR
IC50=6299 nM
IC50=34.67 nM
Cell viability (%)
Log10(Osimertinib) (nM)
C
HCC827/SEN
HCC827/OR
IC50=5094 nM
IC50=22.71 nM
Cell viability (%)
Log10(Osimertinib) (nM)
D
Resistant cells
Parental cells
H1975
HCC827
E
H1975/SEN
H1975/OR
HCC827/SEN
HCC827/OR
F
OE-ctrl or
OE-lncLUCAT1 Cells
DMSO or
Osimertinib
Tumor
Challenge
Intragastric Administration and
tumor volume measurement
Treatment
Tumor
Harvest
Day 0
Random Grouping
Avg size: ~200mm3
Day 6
Day 21

Supplementary Figure 3 related to Figure 3.

(A) A schematic diagram of the experimental design used to establish the osimertinib resistant (OR) LUAD cells (H1975/OR and HCC827/OR).

(B, C) Dose-response curves of osimertinib-resistant and parental cells upon osimertinib treatment at indicated concentrations for 48 hours (n = 3).

(C) Representive images of resistant cells and parental cells. Scale bar, 200 μm.

(D) Colony formation assays of osimertinib-resistant and parental cells treated with osimertinib (5 μM) for 2 weeks (n=3).

(E) A schematic diagram of the experimental design used to establish the LUCAT1-overexpressing animal model.

A

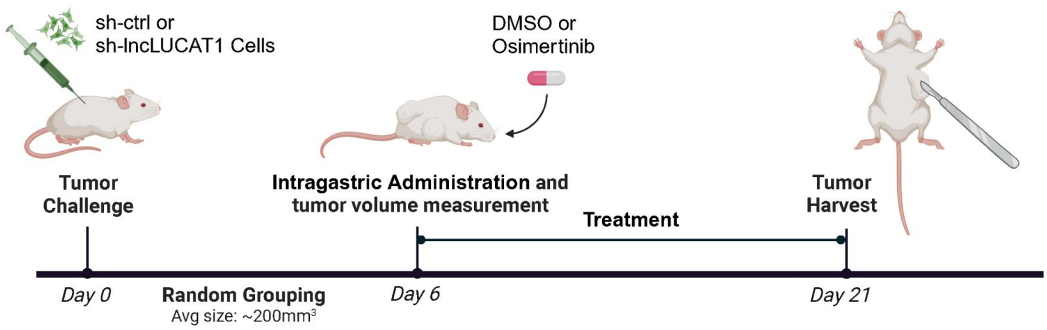

B

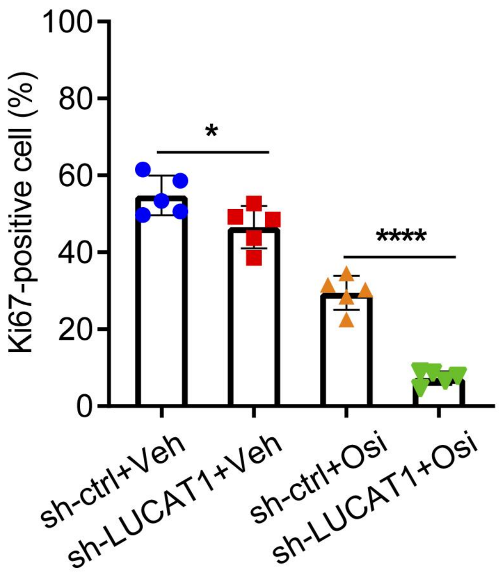

C

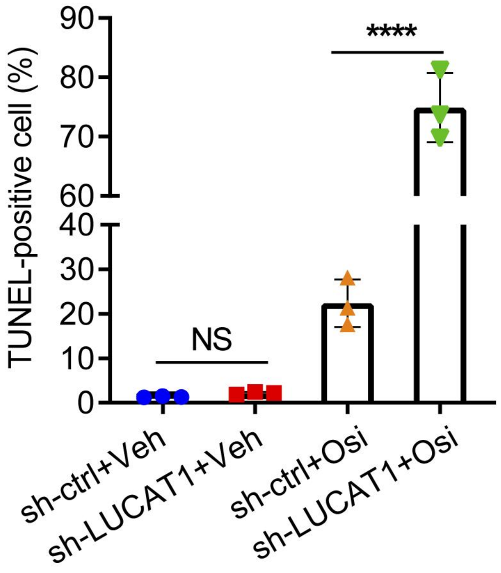

Supplementary Figure 4 related to Figure 4.

(A) A schematic diagram of the experimental design used to establish the LUCAT1-knockdown animal model.

(B) Quantification of IHC data for the positivity of Ki67 in indicated groups was shown. Scale bar, 200 m. Data are represented as the mean ± SD. *P<0.05 and ****P<0.0001, paired t-test, n=5.

(C) Quantification of TUNEL-positive cells in indicated groups was shown. Scale bar, 100 m. Data are represented as the mean ± SD. ****P<0.0001 and NS stands for no significance, paired t-test, n=3.

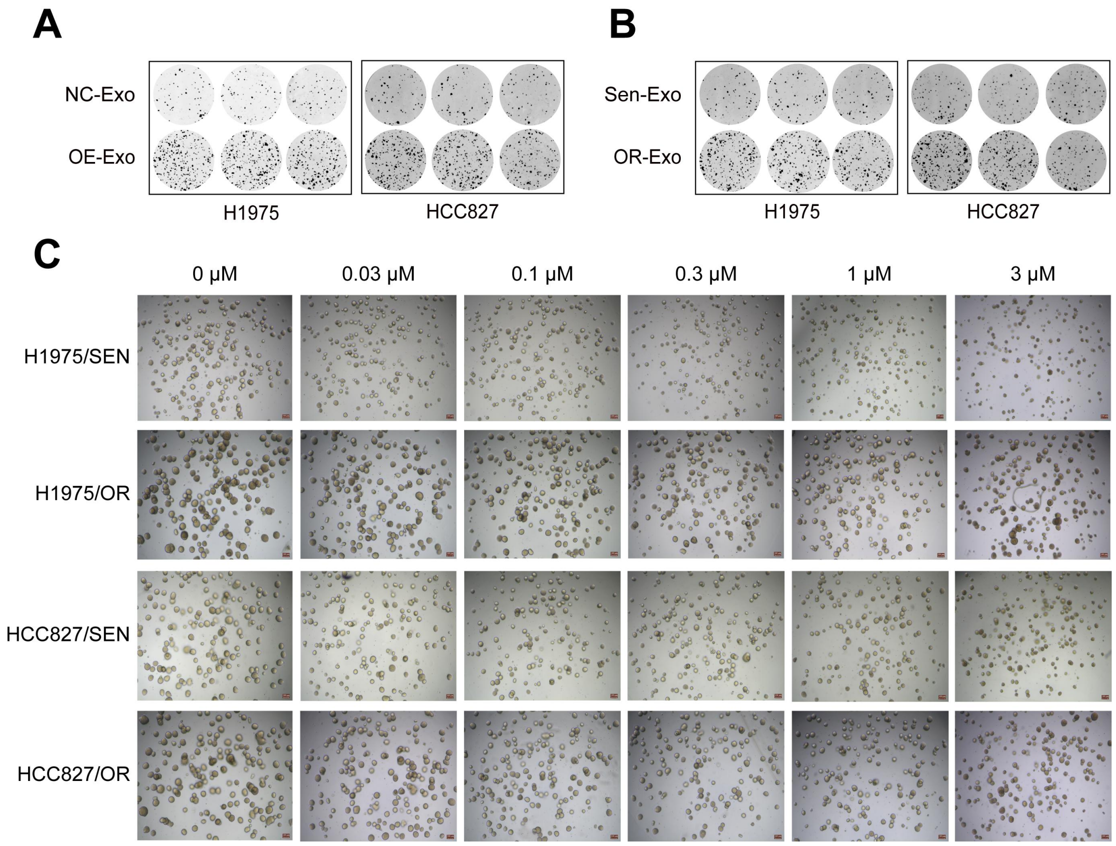

Supplementary Figure 5 related to Figure 5.

(A, B) Colony formation assays of H1975 and HCC827 cells incubated with indicated exosomes with osimertinib treatment at indicated concentrations for 3 weeks (n=3).

(C) Bright-filed images of organoid viability ATP assay of 306# organoid incubated with indicated exosomes (40 μg/ml) with osimertinib treatment at indicated concentrations for 3 days. Scale bar, 100 μm.

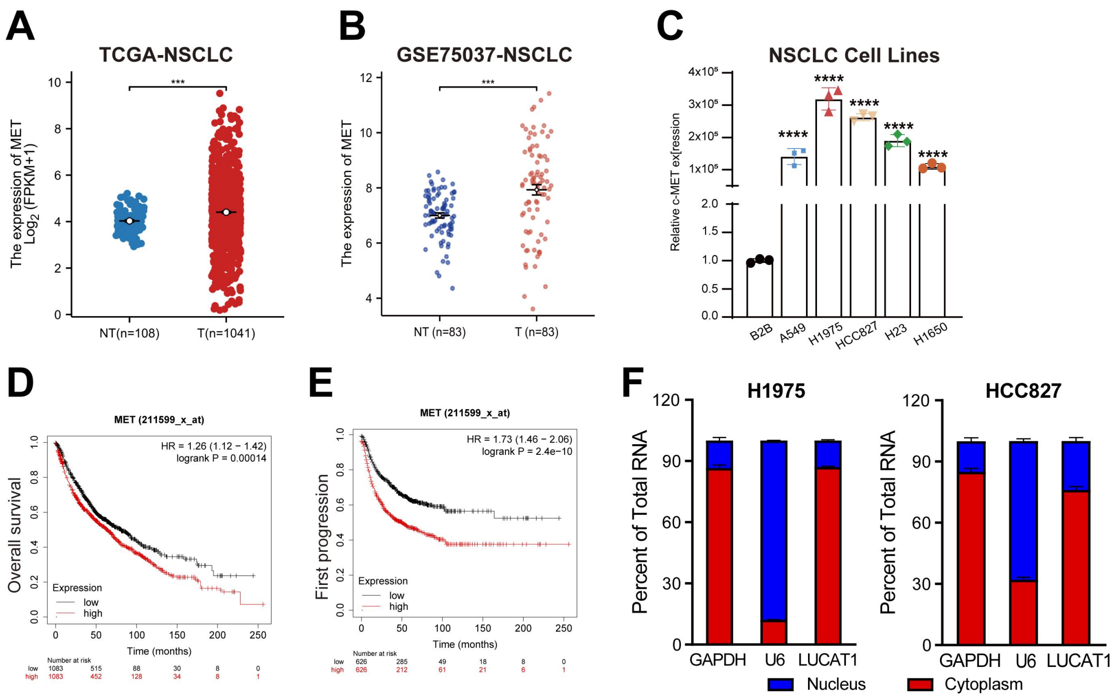

Supplementary Figure 6 related to Figure 6.

(A) Relative expression level of c-MET in adjacent para-tumor tissues (n = 108) and NSCLC tissues (n = 1041) from TCGA cohort. Data are represented as the mean ± SD, ***P<0.001, Wilcoxon rank sum test.

(B) Relative expression level of c-MET in adjacent para-tumor tissues (n = 83) and NSCLC tissues (n = 83) from GSE75037 cohort. Data are represented as the mean ± SD, ***P<0.001, paired t-test.

(C) qRT-PCR analysis of c-MET in B2B cells and indicated NSCLC cell lines. Data are represented as the mean ± SD, ****P<0.0001, one-way ANOVA, n=3.

(D, E) Kaplan–Meier analysis of OS and FP in the high and low c-MET groups according to the median c-MET level in NSCLC patients from TCGA cohort.

(F) Subcellular localization of LUCAT1 in H1975 and HCC827 cells was determined by nucleus-cytoplasm fraction qPCR.

NSCLC, non-small cell lung cancer; TCGA, The Cancer Genome Atlas; qRT-PCR, quantitative real-time polymerase chain reaction; OS, overall survival; FP, first progression.

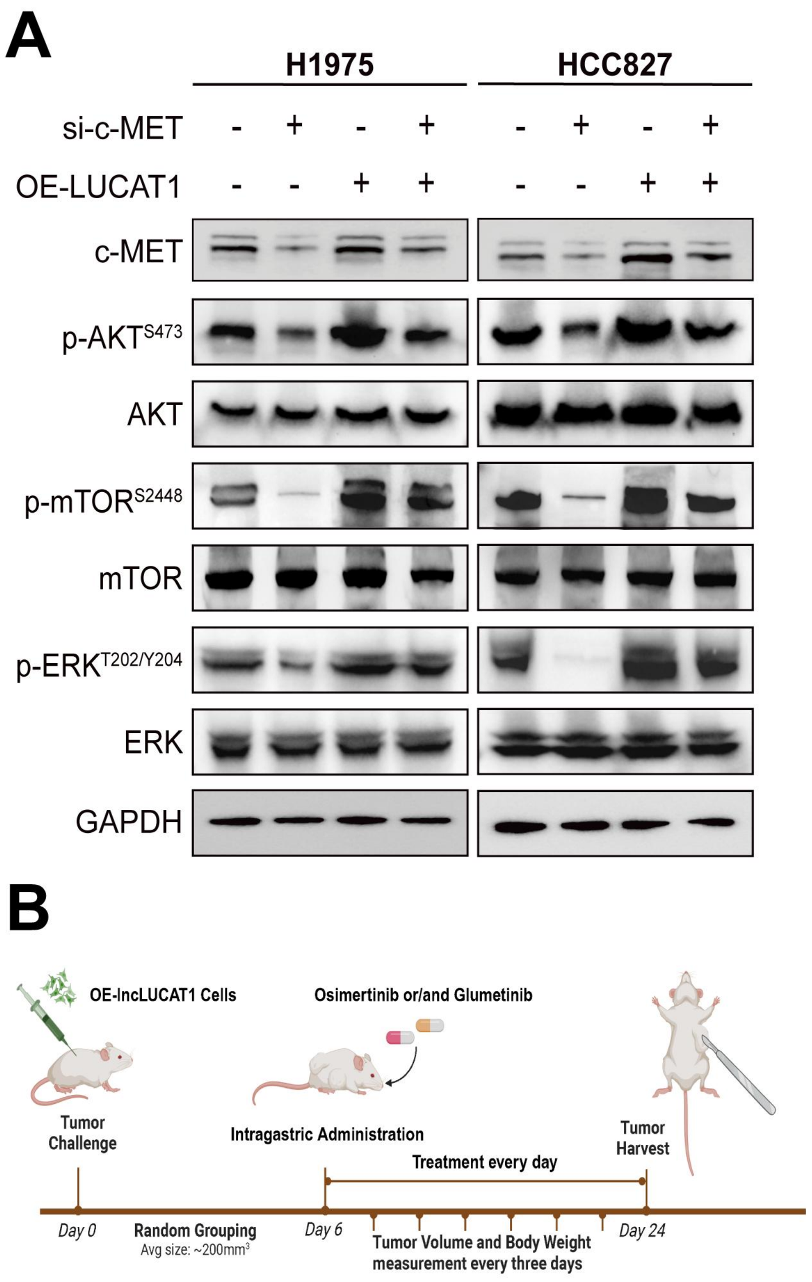

Supplementary Figure 7 related to Figure 7.

(A) Immunoblots of c-MET, p-AKT, AKT, p-mTOR, mTOR, p-ERK, ERK and GAPDH in indicated groups.

(B) A schematic diagram of the experimental design used to establish the animal model. Images of xenograft tumors in indicated groups (n=5 per group).
