# Supplementary material for: Hypoxia-induced exosomal LUCAT1 promotes osimertinib resistance in lung adenocarcinoma by stabilizing c-MET
Source: Cell Death Dis. 2025 Oct 27;16(1):763. doi: 10.1038/s41419-025-08100-2 (PMC12559404; doi:10.1038/s41419-025-08100-2)

Figure 1

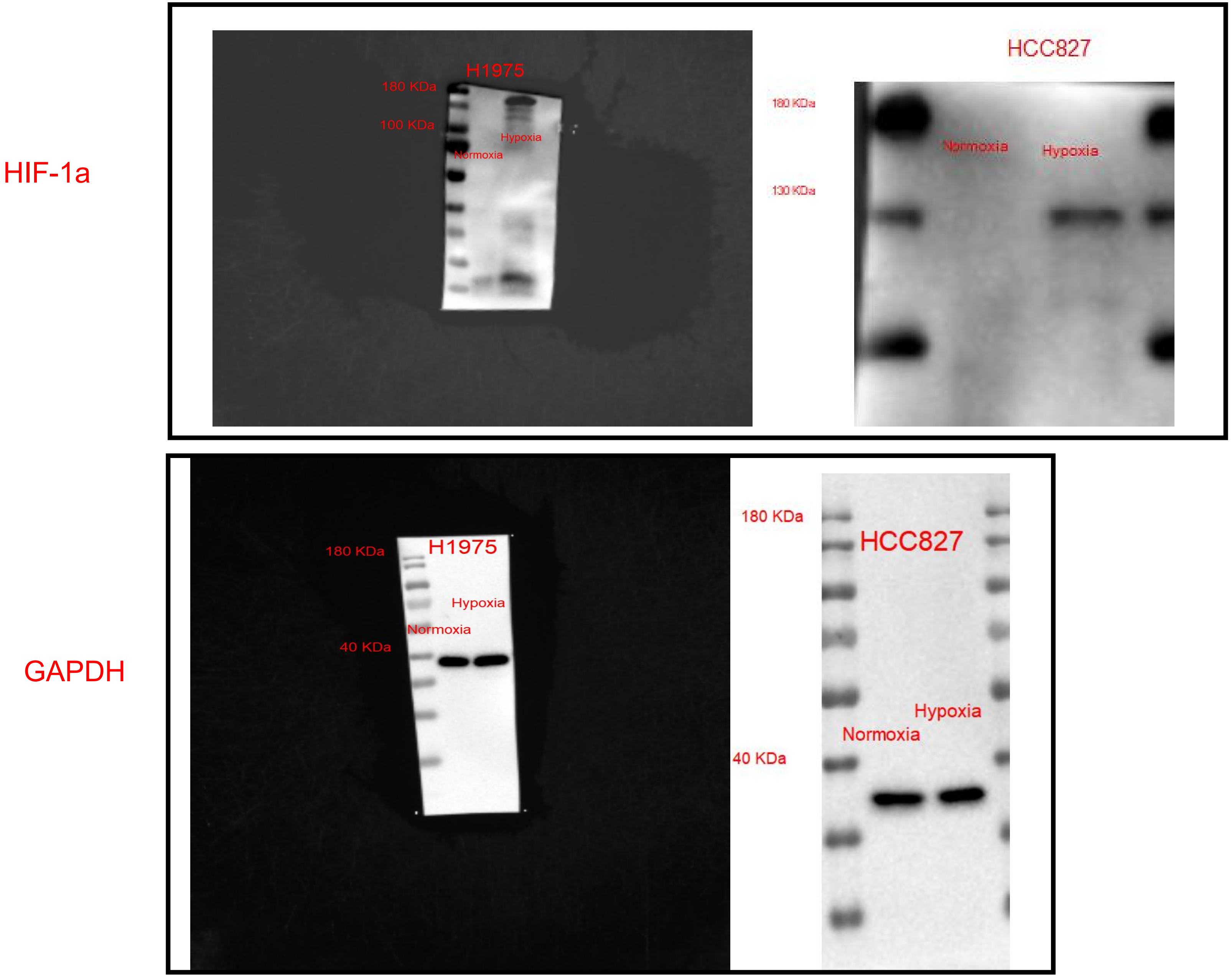
HIF-1a
H1975
180 KDa
100 KDa
Hypoxia
Normoxia
HCC827
180 KDa
130 KDa
Normoxia
Hypoxia
GAPDH
180 KDa
H1975
Hypoxia
Normoxia
40 KDa
180 KDa
HCC827
Hypoxia
Normoxia
40 KDa

Figure 1

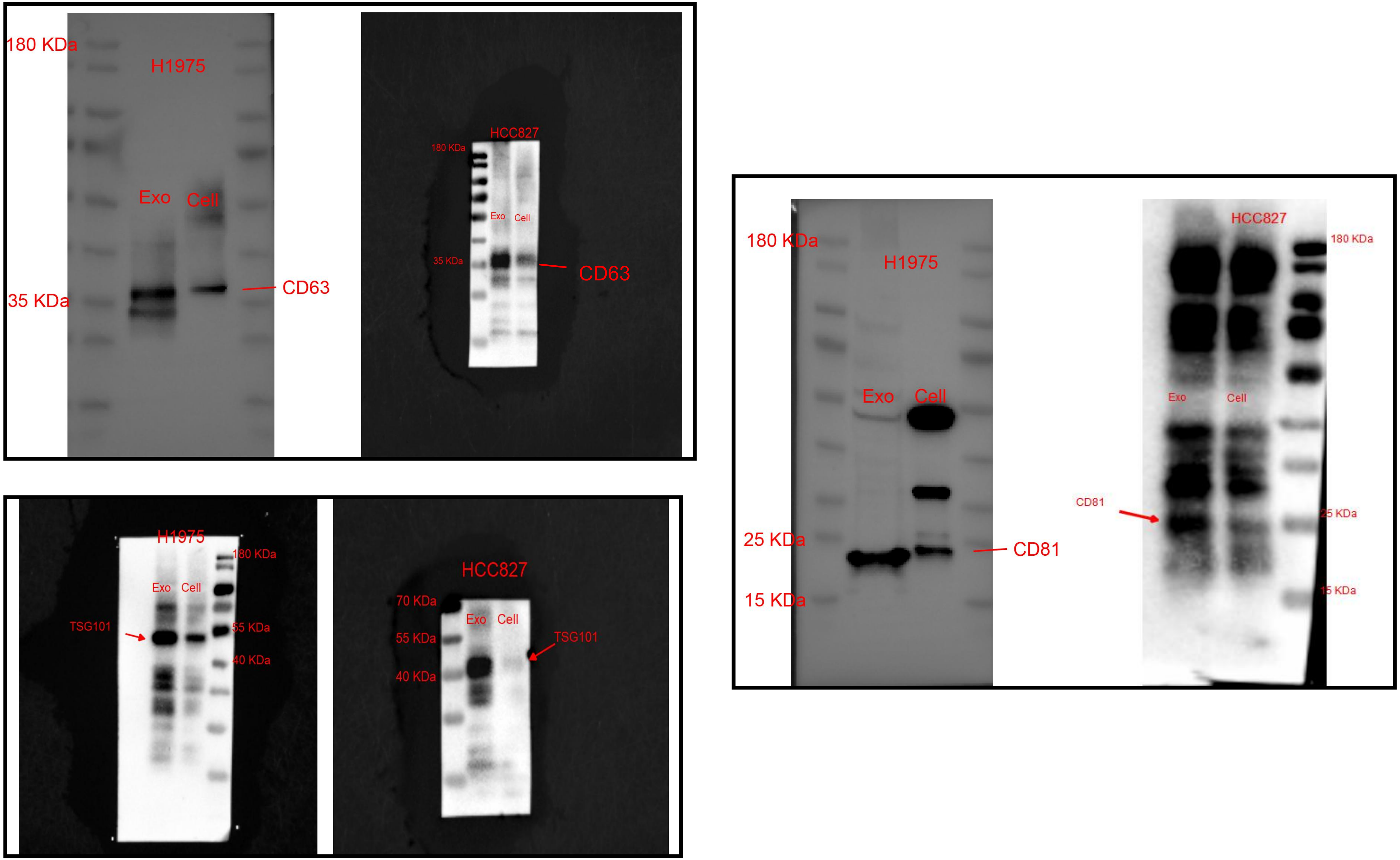
180 KDa
H1975
Exo
Cell
35 KDa
CD63
HCC827
180 KDa
Exo
Cell
35 KDa
CD63
180 KDa
H1975
Exo
Cell
25 KDa
15 KDa
CD81
HCC827
180 KDa
Exo
Cell
CD81
25 KDa
15 KDa
H1975
180 KDa
Exo
Cell
TSG101
55 KDa
40 KDa
HCC827
70 KDa
55 KDa
40 KDa
Exo
Cell
TSG101

Figure 3

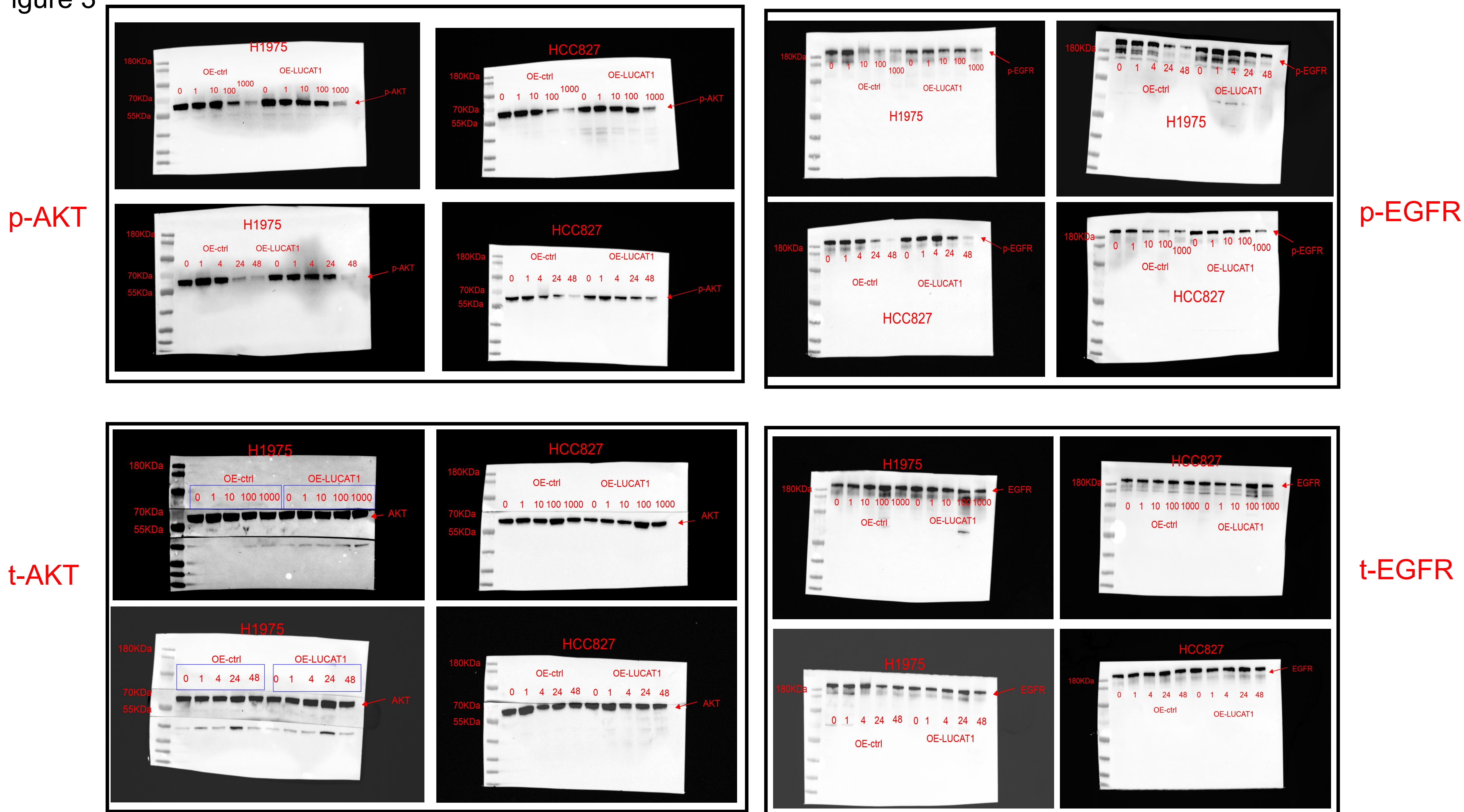
p-AKT
t-AKT
p-EGFR
t-EGFR
H1975
HCC827
OE-ctrl
OE-LUCAT1
180KDa
70KDa
55KDa
p-AKT
AKT
p-EGFR
EGFR
0 1 10 100 1000
0 1 4 24 48

Figure 3

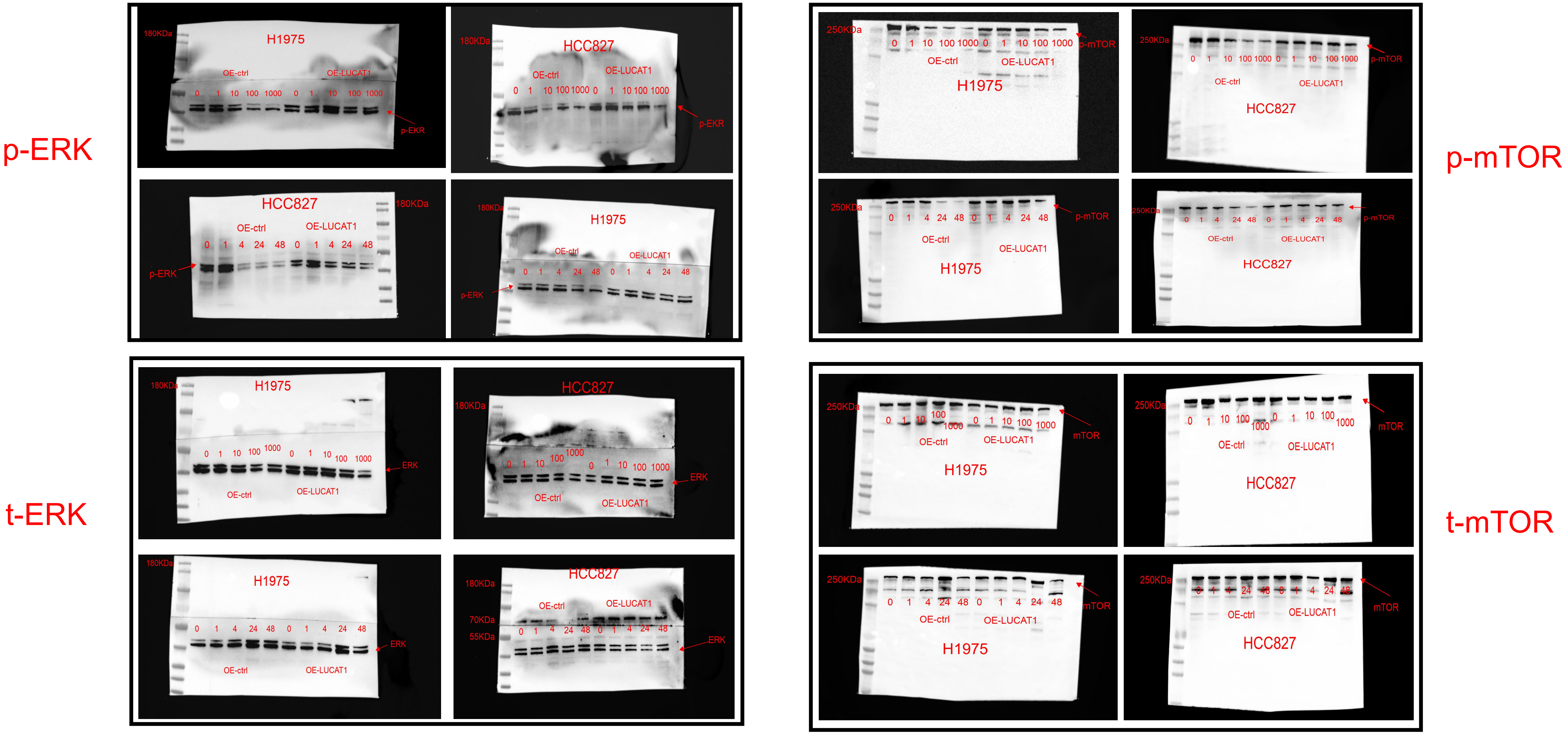
p-ERK
t-ERK
p-mTOR
t-mTOR
H1975
HCC827
OE-ctrl
OE-LUCAT1
180KDa
250KDa
70KDa
55KDa
p-EKR
p-ERK
ERK
p-mTOR
mTOR
0 1 10 100 1000 0 1 10 100 1000
0 1 4 24 48 0 1 4 24 48

Figure 3

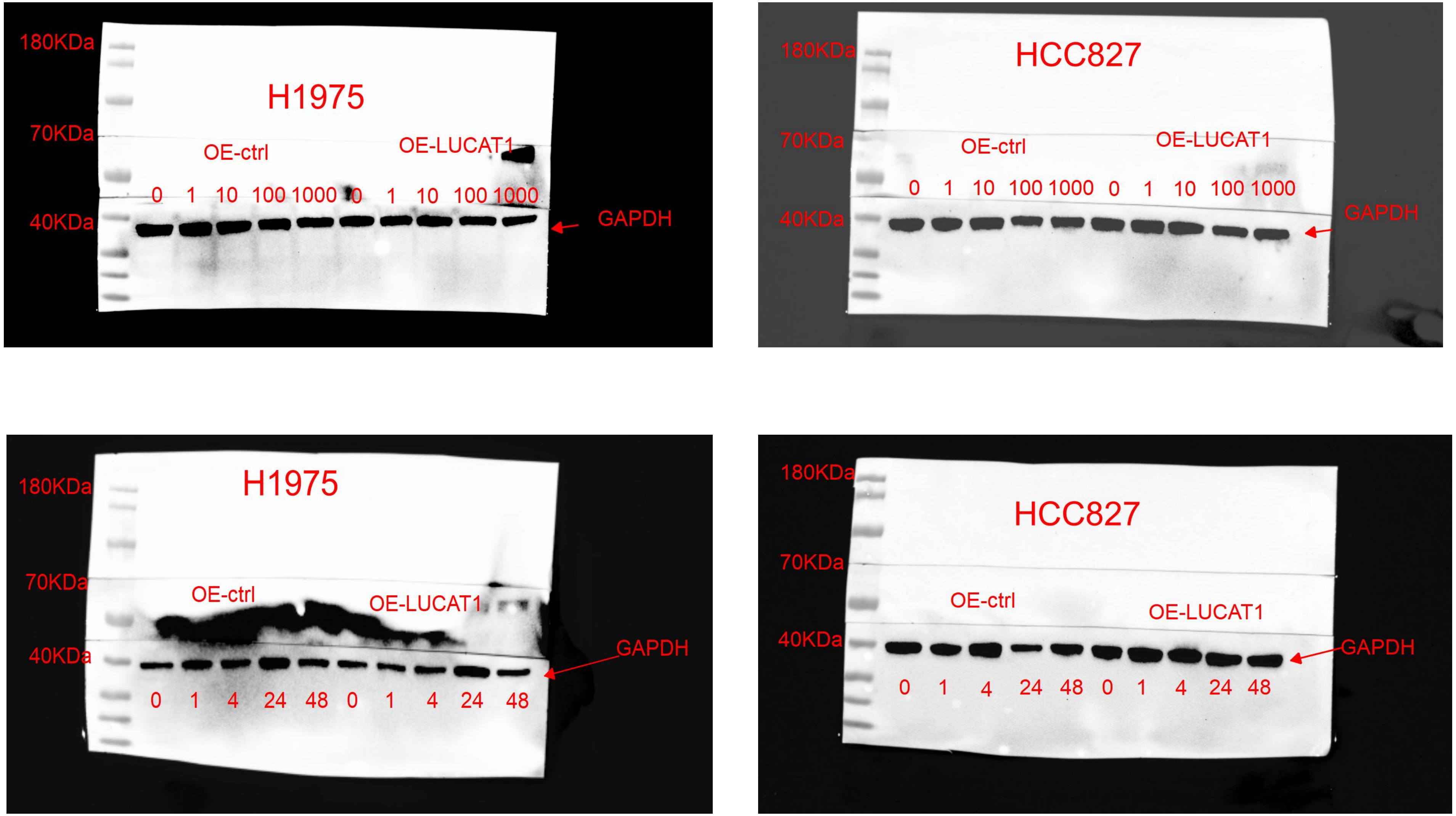
H1975
180KDa
70KDa
40KDa
OE-ctrl
OE-LUCAT1
0 1 10 100 1000 0 1 10 100 1000
GAPDH
HCC827
180KDa
70KDa
40KDa
OE-ctrl
OE-LUCAT1
0 1 10 100 1000 0 1 10 100 1000
GAPDH
H1975
180KDa
70KDa
40KDa
OE-ctrl
OE-LUCAT1
GAPDH
0 1 4 24 48 0 1 4 24 48
HCC827
180KDa
70KDa
40KDa
OE-ctrl
OE-LUCAT1
GAPDH
0 1 4 24 48 0 1 4 24 48

Figure 6

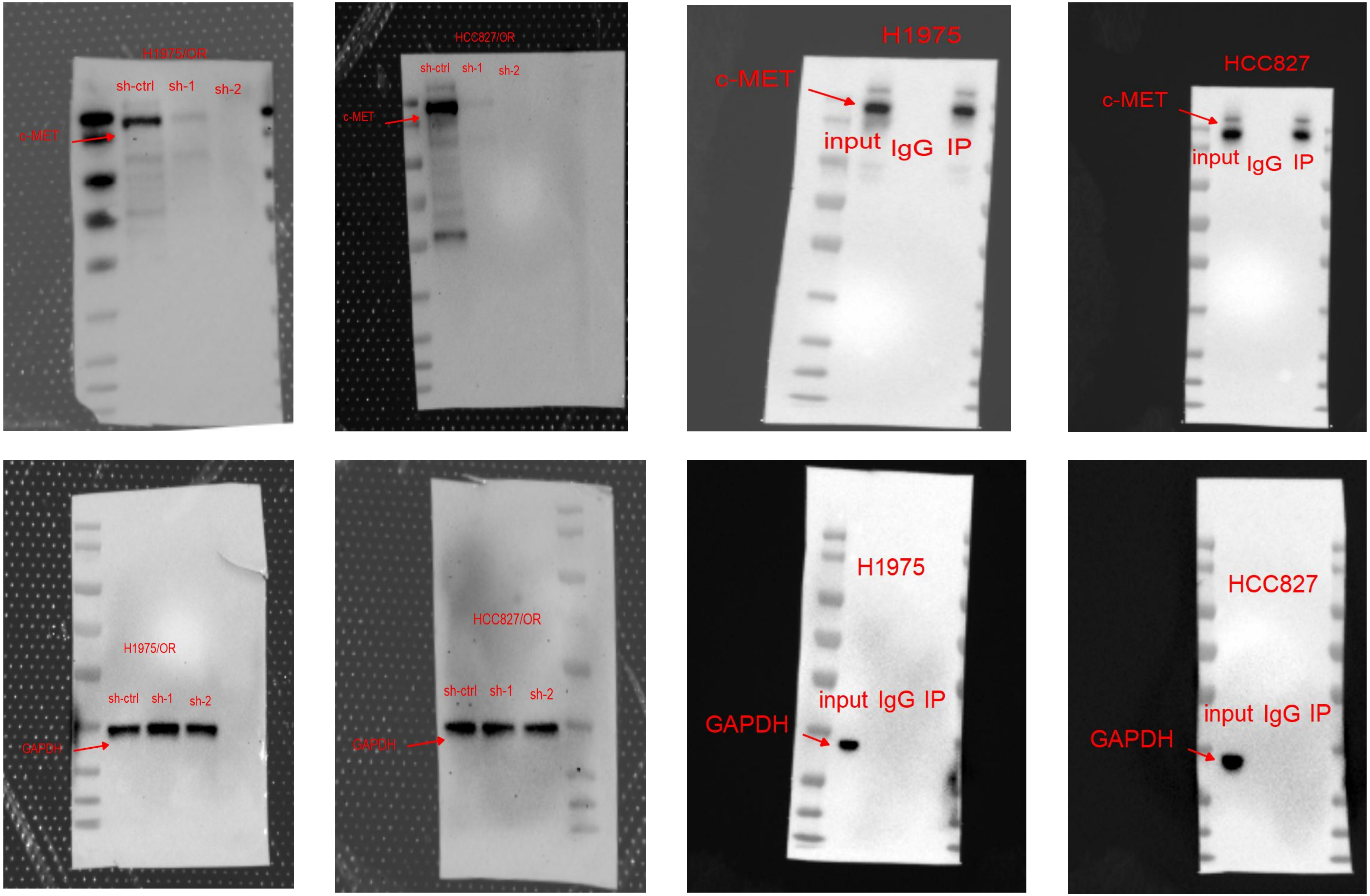
H1975/OR
sh-ctrl sh-1 sh-2
c-MET
HCC827/OR
sh-ctrl sh-1 sh-2
c-MET
H1975
c-MET
input IgG IP
HCC827
c-MET
input IgG IP
H1975/OR
sh-ctrl sh-1 sh-2
GAPDH
HCC827/OR
sh-ctrl sh-1 sh-2
GAPDH
H1975
input IgG IP
GAPDH
HCC827
input IgG IP
GAPDH

Figure 6

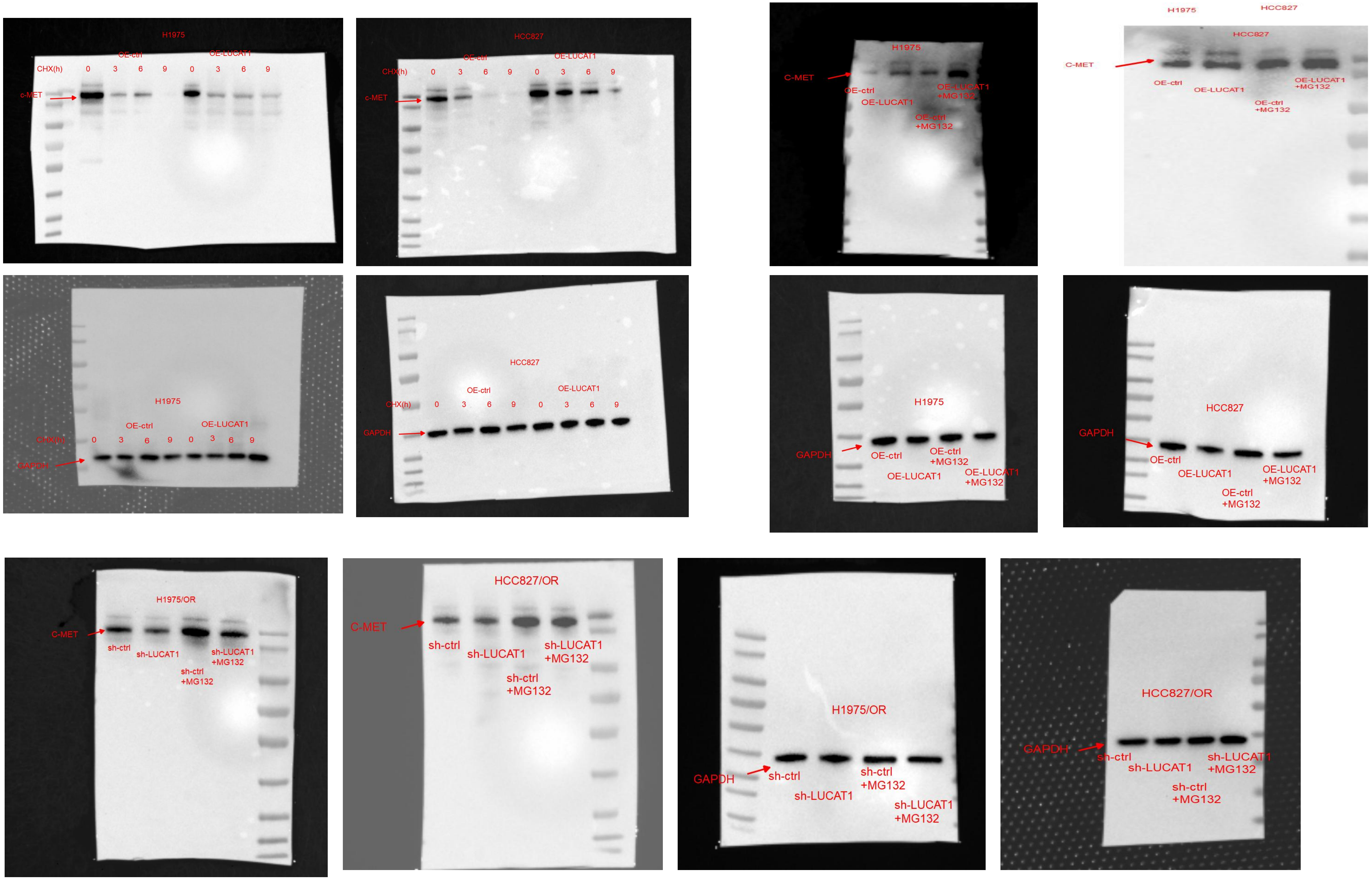
H1975
OE-ctrl
OE-LUCAT1
CHX(h)
0 3 6 9 0 3 6 9
c-MET
HCC827
GAPDH
C-MET
OE-ctrl+MG132
OE-LUCAT1+MG132
H1975/OR
HCC827/OR
sh-ctrl
sh-LUCAT1
sh-ctrl+MG132
sh-LUCAT1+MG132

Figure 6

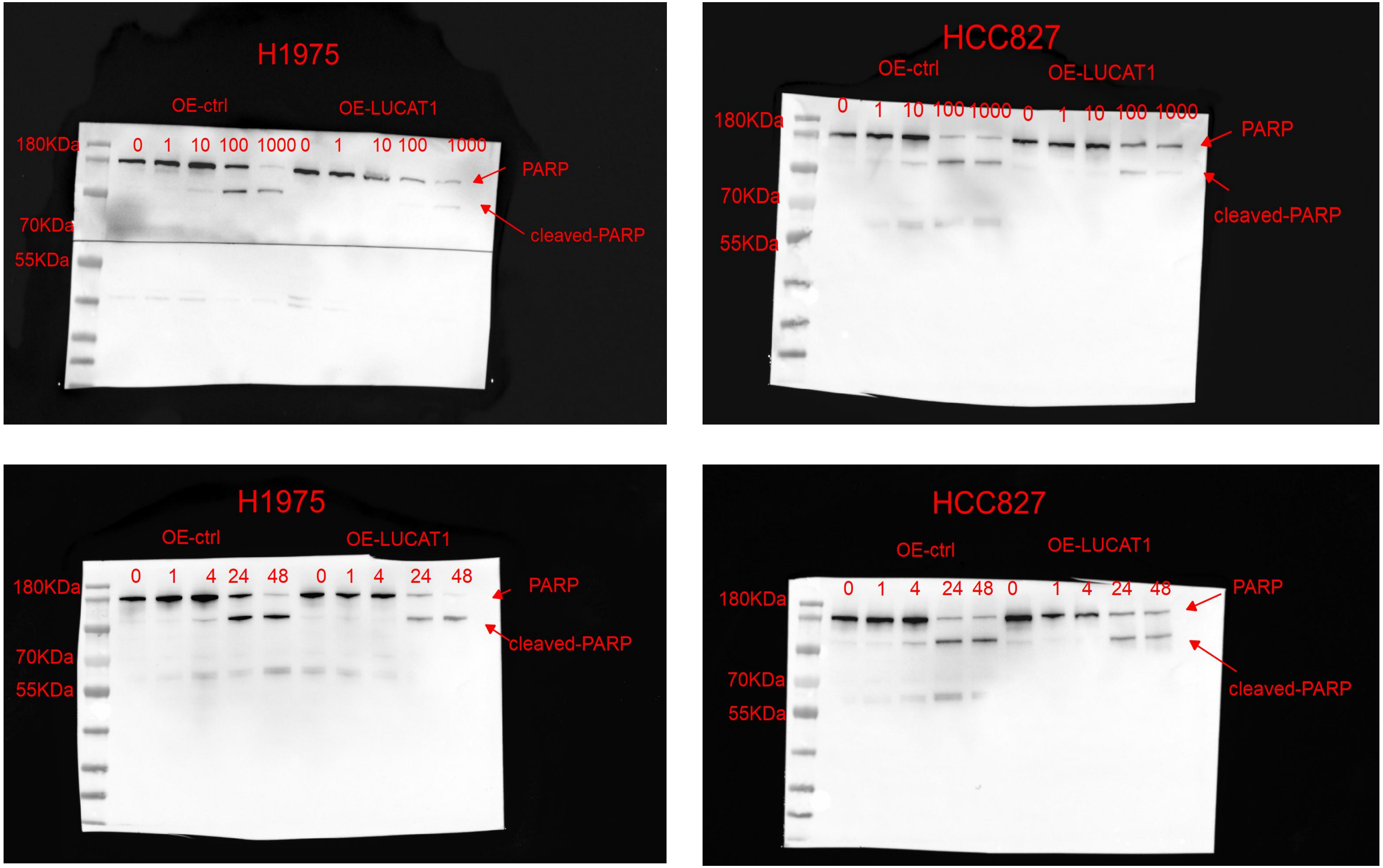
H1975
OE-ctrl
OE-LUCAT1
180KDa
0 1 10 100 1000 0 1 10 100 1000
PARP
70KDa
cleaved-PARP
55KDa
HCC827
OE-ctrl
OE-LUCAT1
180KDa
0 1 10 100 1000 0 1 10 100 1000
PARP
70KDa
cleaved-PARP
55KDa
H1975
OE-ctrl
OE-LUCAT1
180KDa
0 1 4 24 48 0 1 4 24 48
PARP
cleaved-PARP
70KDa
55KDa
HCC827
OE-ctrl
OE-LUCAT1
180KDa
0 1 4 24 48 0 1 4 24 48
PARP
70KDa
cleaved-PARP
55KDa

supFigure 6

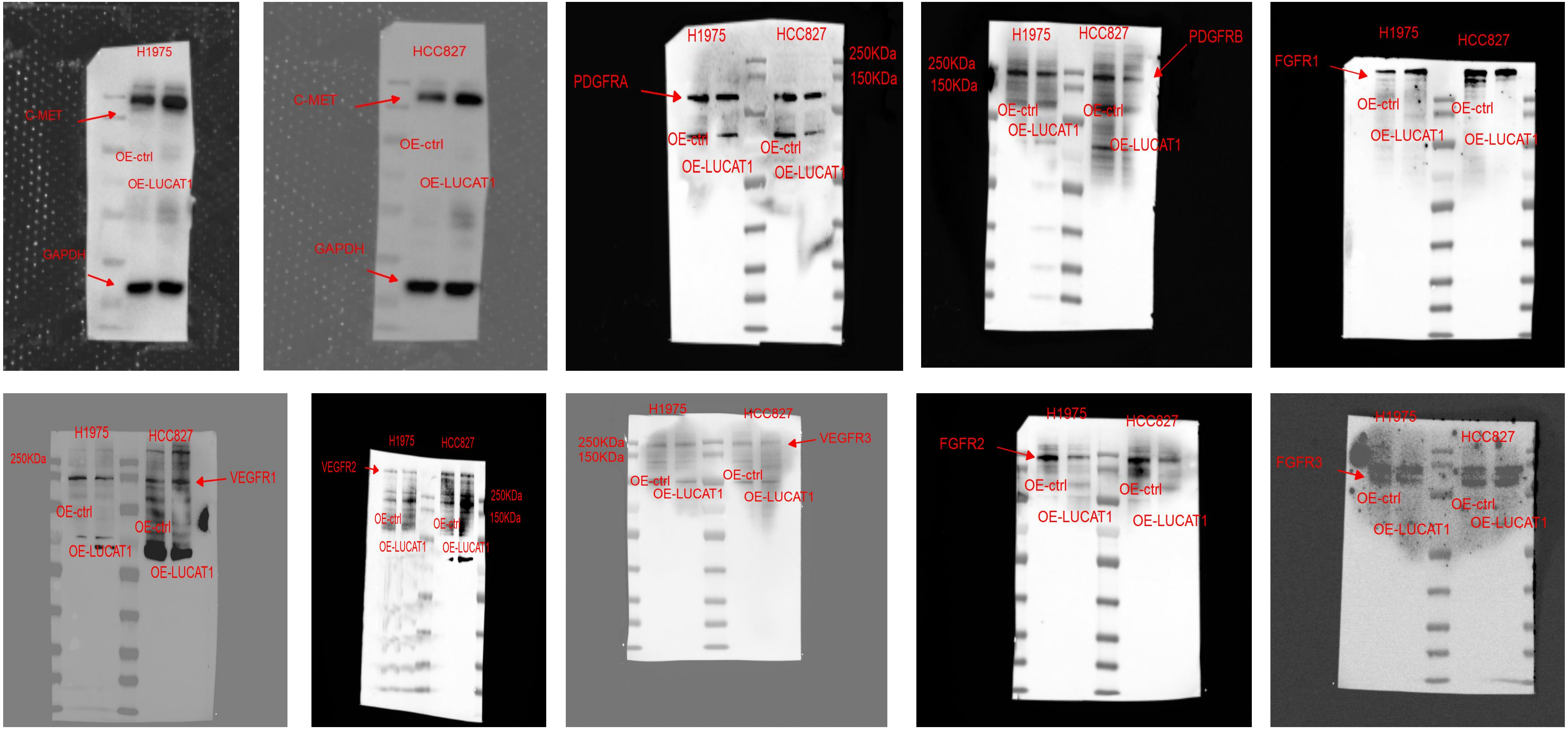
H1975
C-MET
OE-ctrl
OE-LUCAT1
GAPDH
HCC827
C-MET
OE-ctrl
OE-LUCAT1
GAPDH
H1975
HCC827
250KDa
150KDa
PDGFRA
OE-ctrl
OE-LUCAT1
OE-ctrl
OE-LUCAT1
H1975
HCC827
PDGFRB
250KDa
150KDa
OE-ctrl
OE-LUCAT1
OE-ctrl
OE-LUCAT1
H1975
HCC827
FGFR1
OE-ctrl
OE-LUCAT1
OE-ctrl
OE-LUCAT1
H1975
HCC827
250KDa
VEGFR1
OE-ctrl
OE-LUCAT1
OE-ctrl
OE-LUCAT1
H1975
HCC827
VEGFR2
250KDa
150KDa
OE-ctrl
OE-LUCAT1
OE-ctrl
OE-LUCAT1
H1975
HCC827
250KDa
150KDa
VEGFR3
OE-ctrl
OE-LUCAT1
OE-ctrl
OE-LUCAT1
H1975
HCC827
FGFR2
OE-ctrl
OE-LUCAT1
OE-ctrl
OE-LUCAT1
H1975
HCC827
FGFR3
OE-ctrl
OE-LUCAT1
OE-ctrl
OE-LUCAT1

Figure 7

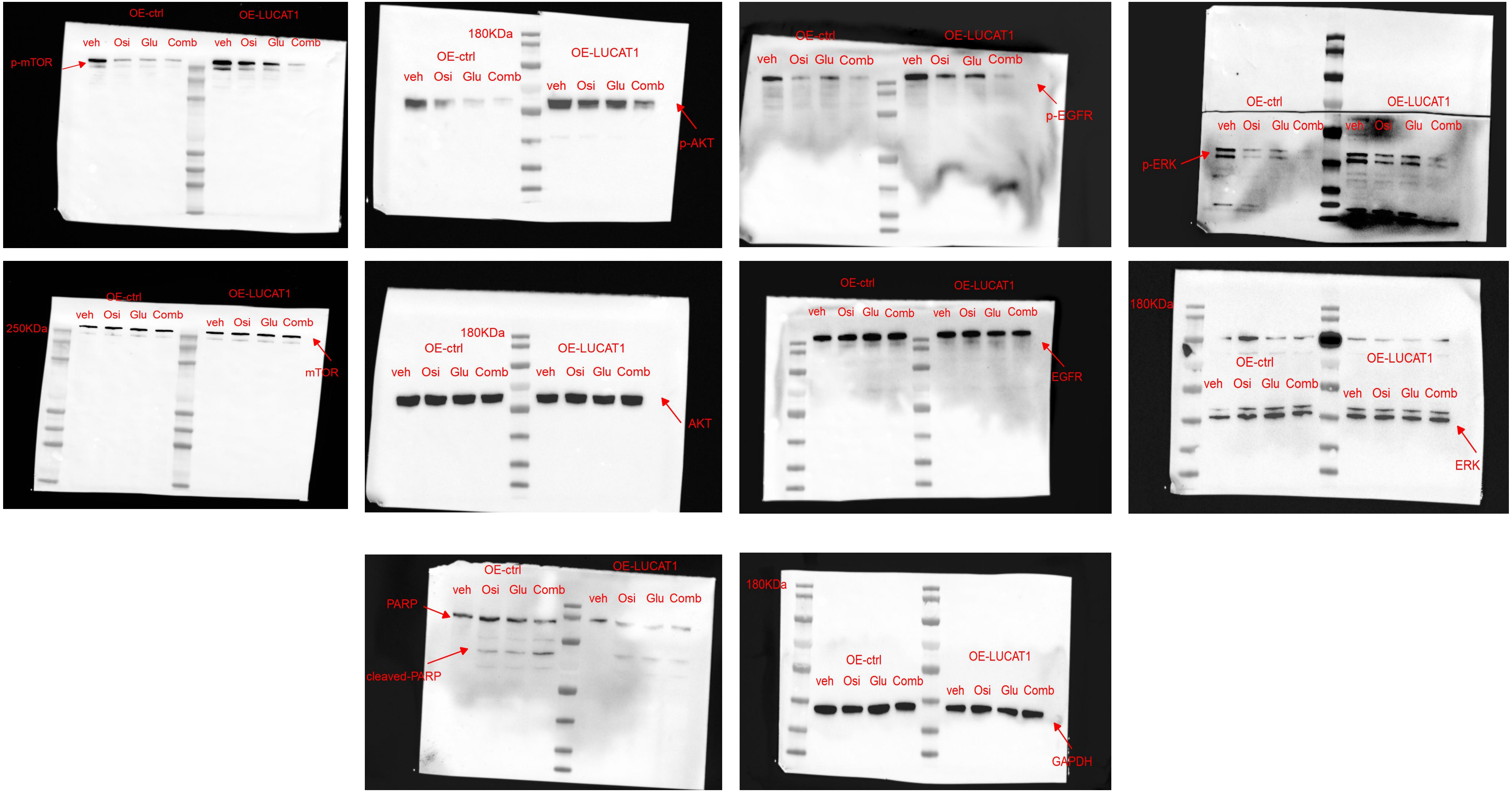
OE-ctrl
OE-LUCAT1
veh
Osi
Glu
Comb
p-mTOR
180KDa
p-AKT
p-EGFR
p-ERK
250KDa
mTOR
AKT
EGFR
ERK
PARP
cleaved-PARP
GAPDH

Figure 8

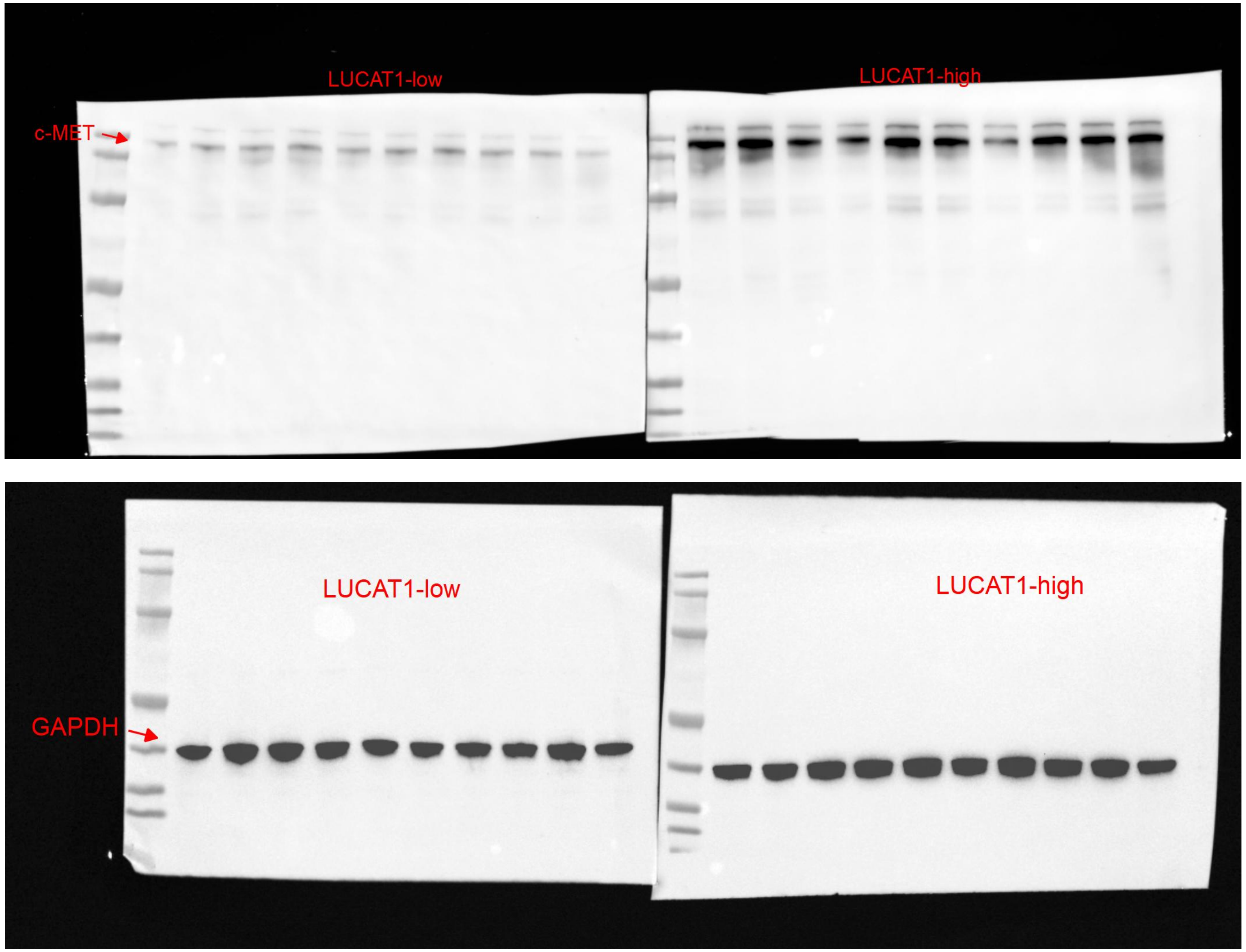

Supplement: Supplementary file 2 — Western Blot_Full unedited gel [file 41419_2025_8100_MOESM2_ESM.pdf]
